# The branched-chain amino acid aminotransferase TaBCAT1 modulates amino acid metabolism and positively regulates wheat rust susceptibility

**DOI:** 10.1093/plcell/koab049

**Published:** 2021-02-05

**Authors:** Pilar Corredor-Moreno, Francesca Minter, Phoebe E Davey, Eva Wegel, Baldeep Kular, Paul Brett, Clare M Lewis, Yvie M L Morgan, Luis A Macías Pérez, Andrey V Korolev, Lionel Hill, Diane G O Saunders

**Affiliations:** 1 John Innes Centre, Norwich Research Park, Norwich, UK; 2 Aix Marseille Université, CNRS, IRD, College de France, CEREGE, Aix-en-Provence, France

## Abstract

Plant pathogens suppress defense responses to evade recognition and promote successful colonization. Although identifying the genes essential for pathogen ingress has traditionally relied on screening mutant populations, the post-genomic era provides an opportunity to develop novel approaches that accelerate identification. Here, RNA-seq analysis of 68 pathogen-infected bread wheat (*Triticum aestivum*) varieties, including three (Oakley, Solstice and Santiago) with variable levels of susceptibility, uncovered a branched-chain amino acid aminotransferase (termed TaBCAT1) as a positive regulator of wheat rust susceptibility. We show that *TaBCAT1* is required for yellow and stem rust infection and likely functions in branched-chain amino acid (BCAA) metabolism, as *TaBCAT1* disruption mutants had elevated BCAA levels. *TaBCAT1* mutants also exhibited increased levels of salicylic acid (SA) and enhanced expression of associated defense genes, indicating that BCAA regulation, via *TaBCAT1*, has a key role in SA-dependent defense activation. We also identified an association between the levels of BCAAs and resistance to yellow rust infection in wheat. These findings provide insight into SA-mediated defense responses in wheat and highlight the role of BCAA metabolism in the defense response. Furthermore, *TaBCAT1* could be manipulated to potentially provide resistance to two of the most economically damaging diseases of wheat worldwide.

## Teaser

A branched-chain amino acid aminotransferase is essential for progression of wheat yellow and stem rust, two of the most economically damaging diseases of wheat worldwide.

## Introduction

Plant pathogens pose a continuous threat to agricultural productivity. Upon pathogen invasion, plants activate a suite of basal defense responses, with locally triggered resistance responses rapidly spreading beyond the infection site. This systemic spread leads to systemic acquired resistance (SAR) or defense priming (Sticher et al., 1997; Conrath, 2011). SAR activation involves the phytohormone salicylic acid (SA; Vlot et al., 2009), the levels of which tightly correspond to expression of defense-related genes (Gaffney et al., 1993). Other plant hormones involved in basal defense signaling, such as jasmonates, ethylene, and abscisic acid, act synergistically or antagonistically to SA, depending on the pathogen’s lifestyle (Verhage et al., 2010). However, long-distance signaling of SAR is complex, likely involving several different molecules (Shah and Zeier, 2013), including amino acid-related molecules such as pipecolic acid, derived from lysine, which accumulates to high levels in *Arabidopsis thaliana* leaves challenged with *Pseudomonas syringae* and acts in both a SA-dependent and -independent manner (Navarova et al., 2012; Bernsdorff et al., 2016). Elevation of pipecolic acid levels in *A. thaliana* induces many genes involved in SAR and basal immunity, whilst it also is converted to the metabolite N-hydroxypipecolic acid (NHP), which acts as a critical regulator of SAR activation (Hartmann et al., 2018). However, beyond the NHP catabolic pathway, the precise role of other amino acid metabolic pathways in SAR activation is unclear.

**Figure:**
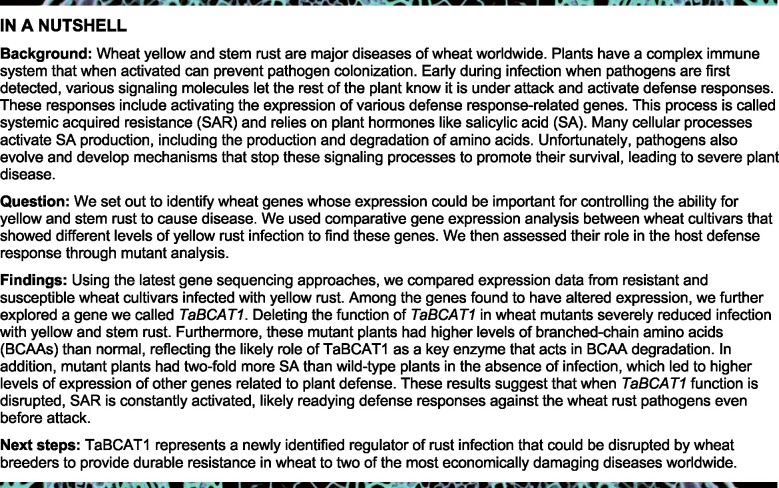


During pathogen invasion, plants produce an array of natural products with antimicrobial properties, and many of these specialized defense molecules are derived from amino acid precursors. The best-known example is the glucosinolates, which act as defense-related secondary metabolites found largely in the Brassicaceae (Ishida et al., 2014). Upon pathogen attack, the hydrolysis of glucosinolates by myrosinases releases various toxic compounds that can directly or indirectly protect plants from fungi, bacteria and insects (Bednarek et al., 2009; Fan et al., 2011). Among the amino acids altered in response to pathogen invasion, branched-chain amino acids (BCAAs) serve as precursors for aliphatic glucosinolates (Sonderby et al., 2010). Plants synthesize BCAAs in chloroplasts and appear to degrade BCAAs across several cellular compartments, including mitochondria (Binder, 2010). The final step in BCAA synthesis is a transamination catalyzed by a BCAA aminotransferase (BCAT). As BCAT is the only enzyme common to both BCAA biosynthesis and degradation, its activity must be carefully modulated to ensure BCAA homeostasis. In *A. thaliana*, seven *BCAT* genes have been identified, with two of the encoded isoenzymes localizing to the chloroplast (AtBCAT-2 and -3), two to the cytosol (AtBCAT-4 and -6), and one to the mitochondria (AtBCAT-1). The localization of AtBCAT-5 is unclear (Diebold et al., 2002) and *At-BCAT-7* is likely a pseudogene (Binder, 2010). BCAA catabolism has been suggested to affect SA/JA crosstalk and may influence responses to pathogen invasion. For instance, silencing of the first enzyme of isoleucine (Ile) biosynthesis in *Nicotiana attenuata* reduced the pool of Ile available for conjugation with JA, which acts to promote resistance to herbivores (Kang et al., 2006). In cereal crops, a *Hordeum vulgare* BCAT gene (*Hvbcat-1*) was identified that encodes a predicted mitochondrial transit peptide (Malatrasi et al., 2006). However, the relationship between BCAAs and immunity in cereals such as barley (*H. vulgare*) and bread wheat (*Triticum aestivum*) is unknown.

Suppression of plant defenses and evasion of recognition are crucial to the survival of obligate biotrophic pathogens such as the wheat yellow and stem rust fungi, *Puccinia striiformis* f. sp. *tritici* (*Pst*) and *Puccinia graminis* f. sp. *tritici* (*Pgt*). These pathogens secrete effectors that manipulate host processes and promote colonization by interacting with specific host target proteins (Bialas et al., 2018). For instance, the biotrophic fungal pathogen *Cladosporium fulvum* secretes the effector Avr2, which directly binds to and inhibits the apoplastic host Cys proteases RCR3 (required for *C. fulvum* resistance) and PIP1 (*Phytophthora*-inhibited protease) in tomato (*Solanum lycopersicum*), suppressing host immunity and thereby enhancing disease susceptibility (van Esse et al., 2008). Certain host genes play essential roles in pathogen colonization, although their biological functions and the effectors that manipulate them remain unknown. For instance, loss of function of the *mildew-resistance locus* (*Mlo*) in barley enhances powdery mildew resistance (Buschges et al., 1997). Although *Mlo* was cloned more than 20 years ago, its biochemical function and the mildew effectors that target it remain elusive. Regardless, the broad, nonrace-specific resistance conferred by loss of *Mlo* function has led to its widespread manipulation in breeding pipelines (Kusch and Panstruga, 2017), illustrating the immense value of elucidating host genes with essential roles in pathogen colonization.

Efforts to identify host genes that are essential for pathogen ingress have largely focused on screening mutant populations or cloning known loci (Pavan et al., 2010). Here, we took an alternative approach, using RNA-seq analysis of *Pst*-infected field-collected wheat samples from varieties with different levels of susceptibility. We reasoned that using multiple samples from the same variety subjected to different environmental conditions and/or at different developmental stages would facilitate the identification of genes linked to *Pst* susceptibility, as the plants’ main commonality was the response to *Pst* infection. Using this approach, we identified *TaBCAT1* as a novel positive regulator of wheat rust susceptibility with a clearly defined function that is required for *Pst* and *Pgt* infection. *TaBCAT1* disruption mutants had elevated levels of BCAAs and SA, and enhanced expression of several pathogenicity-related (*PR*) genes. Taken together, our results suggest that *TaBCAT1*-mediated BCAA regulation has a key role in SA-dependent SAR activation in wheat. *TaBCAT1* thus represents a positive regulator of susceptibility that could potentially be manipulated to provide durable resistance to two of the most economically damaging diseases of wheat worldwide.

## Results

### RNA-seq analysis of *Pst*-infected field samples can be used to define wheat varieties

**Figure 1:**
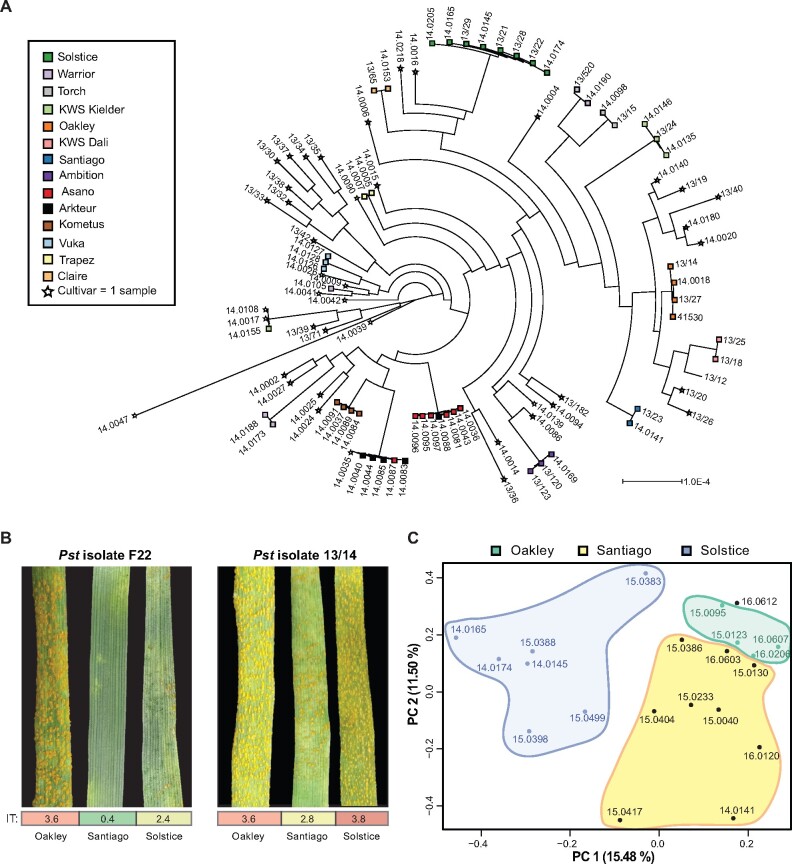
RNA-seq analysis can be used to define the wheat variety in *Pst*-infected wheat samples. A, *Pst*-infected wheat samples cluster genetically based on variety. Phylogenetic analysis was carried out using 96 *Pst*-infected wheat samples with a maximum-likelihood model (227,267,910 nucleotide sites). Scale bar represents nucleotide substitutions per site; colors reflect wheat variety; stars indicate samples from a wheat cultivar with a single sample. Bootstrap values are provided in Supplemental Data File 2. B, The three wheat varieties Oakley, Solstice and Santiago have differing levels of susceptibility to the dominant *Pst* pathotypes (races) in Europe. Each of the three varieties was subjected to *Pst* infection with two isolates (F22 and 13/14) and infection types (IT) recorded 12 dpi following the 0–4 scale (McIntosh et al., 1995). Values represent an average from five independent plants (Supplemental Table 1). C, Principal component (PC) analysis of wheat gene expression profiles illustrates that samples group together by wheat variety, with two well-defined groups: (1) Solstice and (2) Oakley and Santiago (blue, green, and black font, respectively)

A set of 156 *Pst*-infected bread wheat (*T. aestivum*) samples collected between 2013 and 2016 were used to explore the genetic diversity of *Pst*-infected host wheat varieties across Europe (Supplemental Data Set 1). This set included 130 *Pst*-infected samples previously used to study *Pst* population dynamics (Hubbard et al., 2015; Bueno-Sancho et al., 2017). Total RNA was extracted from the remaining 26 samples and subjected to RNA-seq analysis (Supplemental Data Set 1; Hubbard et al., 2015). As each sample gives rise to both wheat and pathogen-derived reads, we determined the proportion of wheat reads by filtering the raw reads from a subset of 131 samples for quality and mapping them to the Chinese Spring wheat reference genome (IWGSC RefSeq v1.0; International Wheat Genome Sequencing et al., 2018), with an average of 33.74 ± 18.59% reads aligned. To assess the degree of *Pst* infection, reads were also independently aligned for all 156 samples to the *Pst* reference genome (isolate PST-130; Cantu et al., 2011; mean 37.39 ± 17.19%; Supplemental Data Set 1). To evaluate the genetic diversity of the host wheat lines, a subset of 96 samples that had greater than 20% reads aligned to the wheat reference genome was selected for phylogenetic analysis with a maximum-likelihood model (227,267,910 nucleotide sites). This analysis illustrated that an RNA-seq-based approach is sufficient to accurately genotype the given wheat varieties, as samples recorded from the same variety tended to cluster together within the phylogeny (Figure 1A;Supplemental Data Files 1 and 2).

The samples were sourced from multiple contributors. To prevent any inaccuracies that may have occurred during sample recording from affecting downstream analysis, we set out to genetically confirm the wheat variety recorded during collection. We analyzed the sequences at a set of 1,831 genetically mapped single nucleotide polymorphism (SNP) positions (Wang et al., 2014). Our samples were recorded as including 68 known wheat varieties (Supplemental Figure 1A), with SNP data available for 21 of the varieties. Of these, 84.84% of samples with ≥20x coverage at enough discriminative SNP sites could be confirmed as the variety of record, with those not confirmed having too few discriminative SNP sites with sufficient coverage and/or likely being mis-classified as a closely related variety (Supplemental Data Set 2). Based on these results, we conclude that RNA-seq data can be used to accurately assess the wheat variety in *Pst*-infected field samples when discriminate sites have sufficient coverage, which is a pre-requisite when analyzing samples from diverse sources. Overall, the greatest number of samples was from the varieties Santiago (10 samples), Solstice (12 samples), and Oakley (13 samples). Accordingly, samples from these varieties were selected for further analysis.

### Wheat varieties differ in disease susceptibility and gene expression profiles

To establish their *Pst* susceptibility levels, Solstice, Oakley, and Santiago varieties were subjected to seedling infection assays with two UK *Pst* isolates (F22 and 13/14) representative of the dominant genetic groups (races) currently present in Europe (Clusters I and IV; Hubbard et al., 2015) where field samples were sourced. Infection types (ITs) were recorded 12-day post-inoculation (dpi) following the 0–4 scale (McIntosh et al., 1995; Figure 1B;Supplemental Table 1). Oakley displayed full susceptibility to both isolates. Solstice exhibited moderate resistance to *Pst* isolate F22 and almost full susceptibility to *Pst* isolate 13/14, whereas Santiago was resistant to *Pst* isolate F22 and showed moderate susceptibility to *Pst* isolate 13/14. Thus, the three wheat varieties have different levels of susceptibility to the dominant *Pst* pathotypes (races) in Europe and, therefore, are suitable candidates for comparative analysis to identify genes linked to the defense response.

To further evaluate the differences in global gene expression profiles for the given varieties that may reflect differences in their *Pst* susceptibility, we evaluated transcript abundances using 21 samples that had a minimum of 15% reads aligned to the wheat transcriptome and were confirmed as the variety recorded (Supplemental Figure 1B). To account for batch, library preparation and sequencing depth differences, we implemented between-sample normalization of the read count data for 81,161 transcripts. Before normalization, a scatterplot of the first two principal components illustrated that the libraries initially clustered largely by year of collection. To normalize the data, we used an approach known as “remove unwanted variation” (RUV; Risso et al., 2014) and defined libraries from the same variety to reduce the relative log expression (RLE) distribution (Supplemental Figures 2 and 3). After this, a scatterplot of the first two principal components grouped samples by wheat variety, with two well-defined groups: (1) Solstice and (2) Oakley and Santiago (Figure 1C). The grouping of expression profiles for the latter two likely reflects their close genetic relationship with Oakley, a parent of Santiago. This analysis confirms that global gene expression profiles in *Pst*-infected field samples tend to group by wheat variety. As these varieties display differences in *Pst* susceptibility, this enabled us to analyze them for genes displaying varietal-specific expression that could potentially play key roles in disease susceptibility.

### Co-expression cluster analysis reveals varietal-specific gene expression profiles

**Figure 2:**
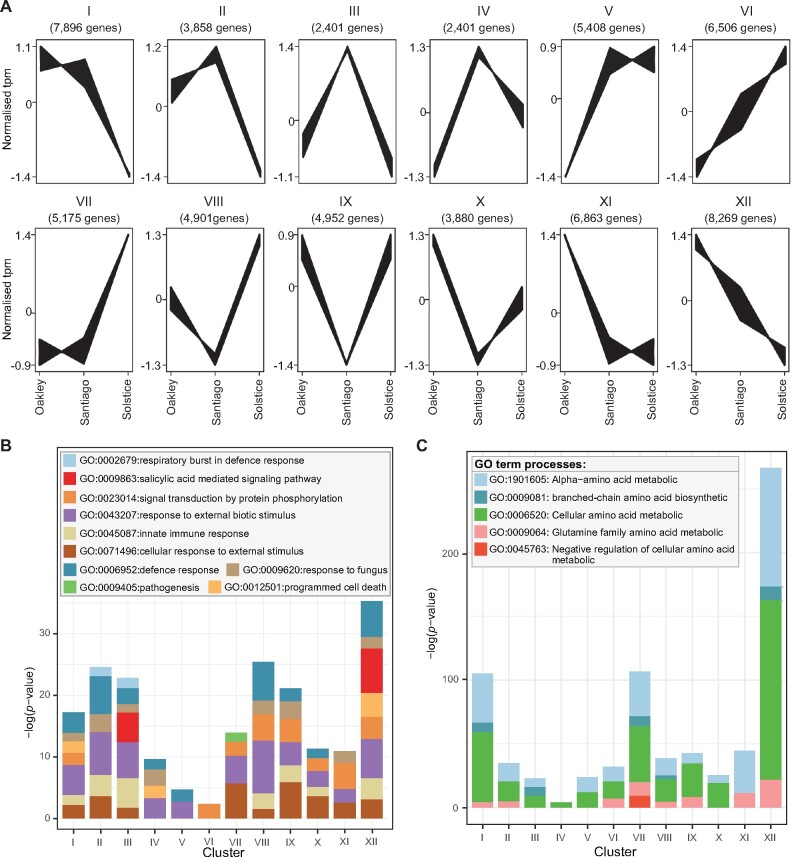
Wheat varieties with different levels of *Pst* susceptibility display variation in expression of genes linked to defense-related processes and amino acid metabolism. A, Gene expression profiling of *Pst*-infected samples identified a total of 61,700 differentially expressed transcripts that were assigned to 12 co-expression clusters (I–XII) that displayed similar patterns of differential expression between the three wheat varieties Oakley, Solstice and Santiago. B, A number of defense-related processes defined in GO terms were enriched across gene expression clusters. C, Processes linked to amino acid metabolism were also enriched across expression clusters. Enrichment was determined based on Fisher’s exact test and GO terms were considered enriched in one cluster at *p* < 0.0001

To reduce dataset complexity and identify biological processes linked to varietal-specific expression, we performed co-expression cluster analysis. As samples from the same wheat variety were sourced from different environmental conditions and were at different developmental stages, we hypothesized that this approach could lead to identification of genes linked to *Pst* susceptibility because the main commonality between samples was their response to *Pst* infection. A total of 61,700 differentially expressed transcripts were identified and classified into 12 co-expression clusters (Figure 2A). Gene ontology (GO) terms were assigned to each gene where possible. This analysis highlighted 16 biological processes, 12 cellular components and 16 molecular functions as second level GO terms that were present in all co-expression clusters (Supplemental Figures 4–6).

To explore functional enrichment between wheat varieties with different levels of susceptibility, we focused on co-expression clusters I, VI, VII, XI, and XII, which displayed the largest differences in gene expression between samples from the most susceptible variety Oakley and the moderately resistant (when inoculated with *Pst* isolate F22) and genetically distinct variety Solstice. Clusters I and XII displayed high levels of expression for the most susceptible variety (gene expression approximately 2.5- and 2.7-fold greater in Oakley than Solstice) and showed the greatest enrichment in defense-related processes, including programmed cell death and innate immune responses (Figure 2B). Additional processes significantly enriched in one or more of the selected clusters (I, VI, VII, XI, and XII) included SA-mediated signaling, photosynthesis, alpha-amino acid and aspartate family amino acid metabolic processes, negative regulation of cellular amino acid metabolism (Figure 2C), and BCAA biosynthetic processes.

**Figure 3:**
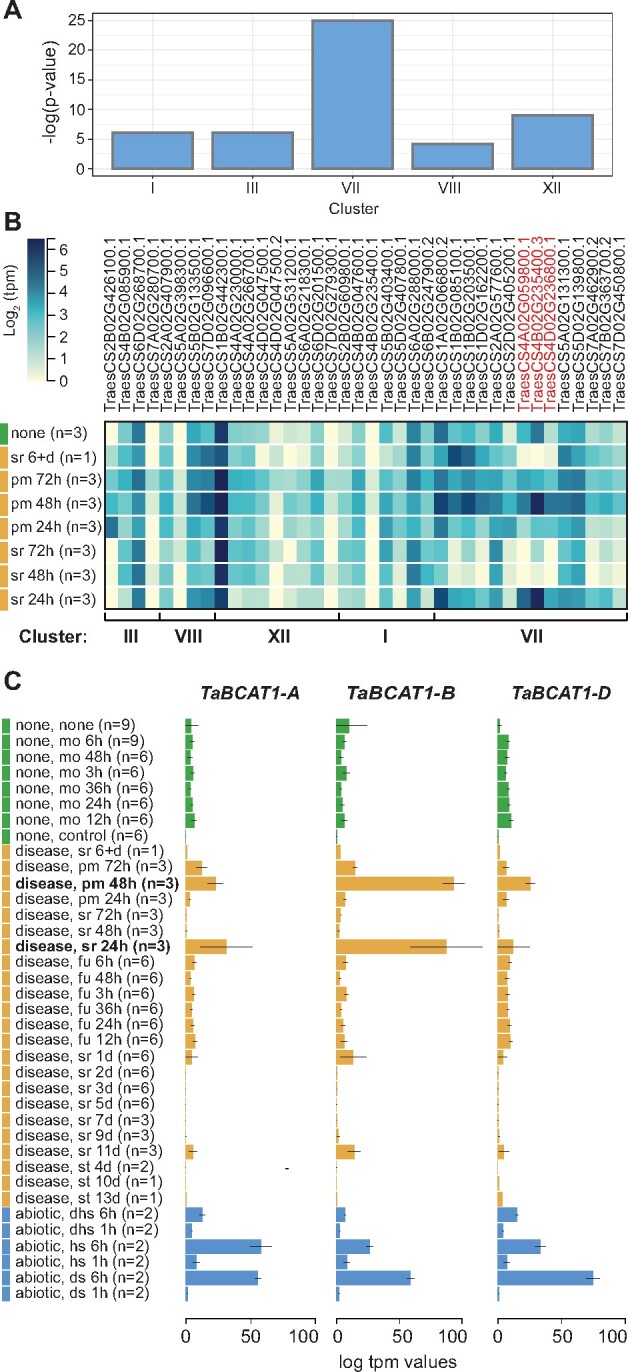
*TaBCAT1* expression is elevated early during infection with *Pst* and *Blumeria graminis* f. sp. *tritici* (*Bgt*). A, A total of 37 transcripts annotated with GO terms linked to BCAA biosynthetic processes were identified across co-expression clusters, with the highest enrichment in cluster VII, followed by cluster XII. B, Gene expression analysis of wheat samples infected with *Pst* and *Blumeria graminis* f. sp. *tritici* (*Bgt*) revealed three gene homeologs (red highlight) uniquely upregulated at 24 hpi and 48 hpi, respectively. C, These three gene homeologs (*TaBCAT1-A*, *TaBCAT1-B*, and *TaBCAT-D*) were upregulated early during infection with *Pst* and *Bgt* and also during abiotic stress. Expression levels for the 37 transcripts were assessed in publicly available wheat RNA-seq data. mo, mock; fu, *Fusarium*; st, *Septoria tritici*; pm, powdery mildew (*Bgt*); sr, stripe rust (*Pst*); hs, heat stress; ds, drought stress; h, hour; d, day; n, number of replicates. Error bars represent standard errors

As amino acid metabolism plays an important role in plant defense responses (Zeier, 2013), we further investigated genes involved in BCAA biosynthetic processes and their expression during infection. A total of 37 transcripts with this function were identified across co-expression clusters, with the highest enrichment in cluster VII, followed by cluster XII (Figure 3A). We analyzed the expression of these 37 transcripts during fungal infection in publicly available wheat RNA-seq data (Borrill et al., 2016). Notably, three gene homeologs (TraesCS4A02G059800.1, TraesCS4B02G235400.3, TraesCS4D02G236800.1) were uniquely upregulated early during infection at 24-h post-inoculation (hpi) in wheat infected with *Pst* and at 48 hpi for wheat infected with *Blumeria graminis* f. sp. *tritici* (*Bgt*; Zhang et al., 2014; Figure 3, B–C). As this pattern could be indicative of a role early in the disease response, we focused on the corresponding gene, annotated as encoding a BCAA aminotransferase that we termed *T. aestivum* branched-chain aminotransferase 1 (TaBCAT1; Supplemental Figure 7). Overall, this analysis identified a number of processes including amino acid metabolism linked to varietal-specific gene expression and highlighted *TaBCAT1* as a candidate disease response gene.

### Disruption of *TaBCAT1* inhibits yellow and stem rust infection

**Figure 4:**
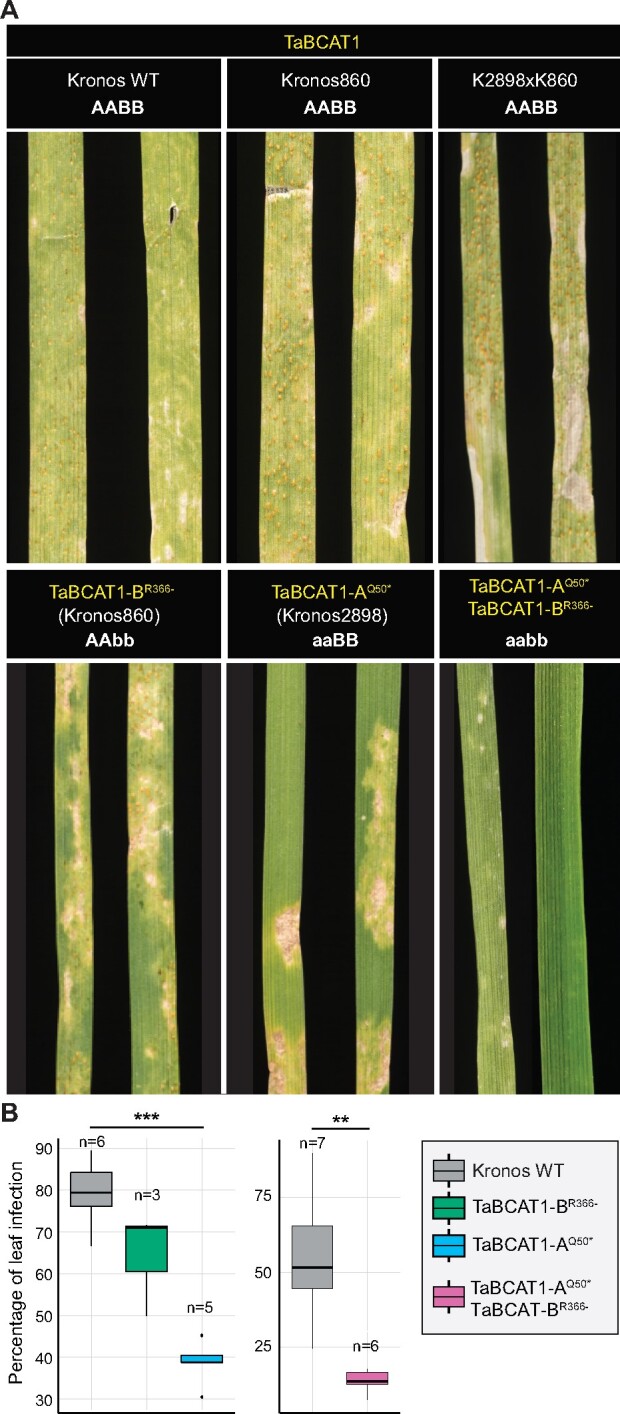
*TaBCAT1* disruption mutants display reduced susceptibility to *Pst*. A, TaBCAT1-A^Q50*^, TaBCAT1-B^R366-^, and TaBCAT1-A^Q50*^ TaBCAT1-B^R366-^ disruption mutants all displayed limited sporulation, higher degrees of necrosis and less chlorosis when infected with *Pst* isolate 13/14 and compared with the Kronos wild-type (WT). Negative controls included Kronos WT and Kronos ethyl methanesulfonate mutants (Kronos860 and K2898xK860) carrying WT alleles of *TaBCAT1*. Images were captured 20 dpi. B, The percentage of leaf infection was significantly reduced in *TaBCAT1* disruption mutants at 20 dpi. Asterisks denote statistically significant differences between each pair of conditions (***p <* *0.001, **p <* *0.01, two-tailed *t*-test). Bars represent median values, boxes signify the upper (Q3) and lower (Q1) quartiles, and whiskers are located at 1.5 the interquartile range

To test whether *TaBCAT1* has a key role in disease progression, in which case its loss of function would result in wheat plants with enhanced susceptibility or resistance to *Pst* infection, we identified tetraploid Kronos TILLING mutants (Krasileva et al., 2017). For the A genome, the Kronos2898 mutant line encoded an early stop codon mutation at amino acid 50 of TaBCAT1, and for the B genome, line Kronos860 had a splice acceptor variant in the last intron, predicted to result in protein truncation at amino acid 366 (Supplemental Figure 8A). Compared to the WT (*cv.* Kronos), F_2_ homozygous single (TaBCAT1-A^Q50*^ and TaBCAT1-B^R366-^) and double mutant lines exhibited differences in lesion size and sporulation level at 20 dpi with *Pst* isolate 13/14 (Hubbard et al., 2015; Figure 4A). All mutant lines supported limited sporulation and showed higher degrees of necrosis and less chlorosis, with the double mutant line (TaBCAT1-A^Q50*^ TaBCAT1-B^R366-^) displaying an almost complete resistant phenotype (Figure 4A). The double mutant line (TaBCAT1-A^Q50*^ TaBCAT1-B^R366-^) and TaBCAT1-A^Q50*^ single mutant had significantly less leaf area infected compared to WT, while the difference was not significant for the TaBCAT1-B^R366-^ single mutant (Figure 4B). By contrast, the negative control (K2898xK860 line carrying WT alleles) displayed a WT phenotype, with high levels of *Pst* sporulation. In conclusion, *TaBCAT1* disruption mutants showed reduced susceptibility to *Pst* isolate 13/14.

To determine if this resistance was specific to *Pst*, we infected the mutant lines with UK *Pgt* isolate UK-01 (Lewis et al., 2018) and scored macroscopic phenotypes at 16 dpi. Kronos had limited susceptibility to UK-01 as evidenced by low levels of sporulation (Supplemental Figure 8, B and C). However, we did observe significantly fewer lesions in both single mutants (TaBCAT1-A^Q50*^ and TaBCAT1-B^R366-^) and the double mutant (TaBCAT1-A^Q50*^ TaBCAT1-B^R366-^); on average, there were 4.4-, 5.7-, and 6-fold reductions in lesions per cm of leaf area in TaBCAT1-A^Q50*^, TaBCAT1-B^R366-^, and the double mutant, respectively. In addition, none of the lesions in TaBCAT1-A^Q50*^ TaBCAT1-B^R366-^ showed sporulation, suggesting these plants had increased resistance to *Pgt*. We conclude that *TaBCAT1* disruption reduced susceptibility to both *Pst* (isolate 13/14) and *Pgt* (isolate UK-01), indicating that TaBCAT1 may play a key role in *Pst* and *Pgt* susceptibility.

### *TaBCAT1* expression is required early during *Pst* infection

**Figure 5:**
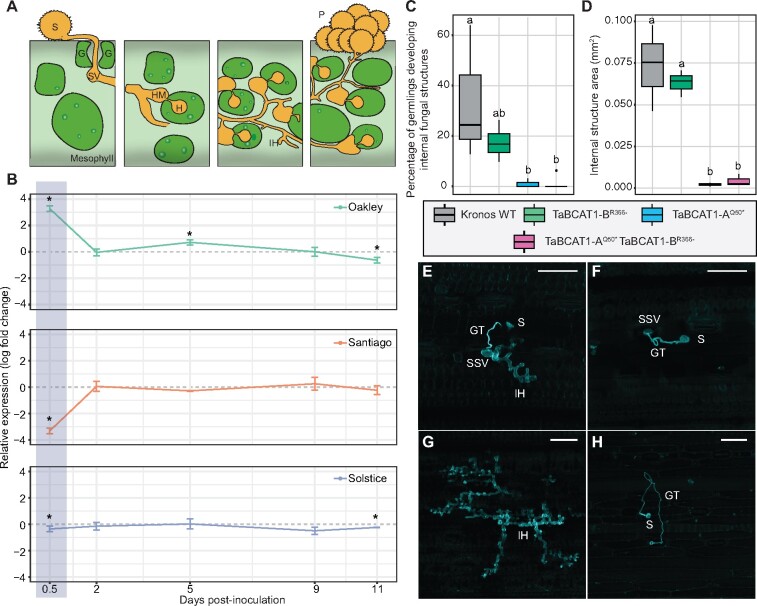
*TaBCAT1* expression early during *Pst* infection is required for susceptibility. A, A controlled time-course of infection was carried out with *Pst* isolate F22 and wheat varieties Oakley, Solstice and Santiago. S, urediniospore; SV, sub-stomatal vesicle; IH, invasive hyphae; HM, haustorial mother cell; H, haustorium; P, pustule; G, guard cell. B, During *Pst* infection, *TaBCAT1* expression at 12 hpi was highest in the most susceptible variety Oakley, whereas the most resistant variety Santiago displayed a significant reduction in *TaBCAT1* expression (highlighted area). Two independent leaves from the same plant were pooled and three independent plants analyzed for *TaBCAT1* expression by RT-qPCR at 12 hpi, 2 dpi, 5 dpi, 9 dpi, and 11 dpi. *TaBCAT1* expression was compared between *Pst*-infected and mock-inoculated plants for each time point per variety. Asterisks denote statistically significant differences (***p <* *0.005, **p <* *0.01, *p <* *0.05; two-tailed *t* test). Error bars represent standard deviations. C, D, Histological studies using a fungus-specific fluorescent dye revealed differences in the extension of internal fungal structures between WT and the TaBCAT1-A^Q50*^ TaBCAT1-B^R366-^ disruption mutant. The number of germinating spores assessed was as follows: Kronos WT *n *=* *229, TaBCAT1-A^Q50*^n =* *114, TaBCAT1-B^R366-^*n *=* *297, TaBCAT1-A^Q50*^ TaBCAT1-B^R366-^*n *=* *152. The number of internal structures measured was as follows: Kronos WT *n *=* *9, TaBCAT1-A^Q50*^n =* *2, TaBCAT1-B^R366-^*n *=* *10, TaBCAT1-A^Q50*^ TaBCAT1-B^R366-^*n *=* *3. Letters indicate significant differences determined using Duncan’s multi range test (*p *<* *0.05). Bars represent median values, boxes signify the upper (Q3) and lower (Q1) quartiles, and whiskers are located at 1.5 the interquartile range. E, WT plants showed clear *Pst* hyphal development at 4 dpi. F, Hyphal growth was not observed for TaBCAT1-A^Q50*^ TaBCAT1-B^R366-^ at 4 dpi. G, At 6 dpi WT plants showed large, intricate fungal structures. H. The TaBCAT1-A^Q50*^ TaBCAT1-B^R366-^ disruption mutant at 6 dpi showed a unique phenotype where germinating spores reached stomata and entered the underlying sub-stomatal space, but further fungal growth was absent. Scale bars in E–H represent 100 µm; S, urediniospore; GT, germ tube; SSV, sub-stomatal vesicle; IH, invasive hyphae

As *TaBCAT1* expression corresponds with the early stages of *Pst* and *Bgt* infection (Zhang et al., 2014), we further evaluated *TaBCAT1* expression during *Pst* infection in the wheat varieties Oakley, Santiago, and Solstice. We carried out RT-qPCR at 12 hpi, 2 dpi, 5 dpi, 9 dpi, and 11 dpi with UK *Pst* isolates F22 and 13/14 using primers amplifying all *TaBCAT1* copies simultaneously and compared expression between infected and mock-inoculated plants (Supplemental Table 2). In plants infected with *Pst* isolate F22, *TaBCAT1* expression was significantly higher at 12 hpi in the most susceptible variety Oakley, showed slightly lower expression in the moderately susceptible variety Solstice and was significantly lower in the most resistant variety Santiago (Figure 5, A and B). When plants were infected with *Pst* isolate 13/14, *TaBCAT1* expression was again significantly higher in the most susceptible variety Oakley (Supplemental Figure 9). In all susceptible interactions, the initial higher level of *TaBCAT1* expression then decreased substantially, by 2 dpi for Oakley infected with *Pst* isolate F22 and 5 dpi for Oakley infected with *Pst* isolate 13/14, respectively. Thus, *TaBCAT1* expression early during infection appears to be linked to the level of *Pst* susceptibility.

We next determined the stage at which *Pst* colonization was perturbed in the TILLING mutant lines. Histological studies with a fungus-specific fluorescent dye (Koch and Pimsler, 1987) at 4 and 6 dpi with *Pst* isolate 13/14 revealed differences in the extension of internal fungal structures between WT and mutant plants (Figure 5, C–H). For instance, 33.72 ± 26.83% of germinating spores in WT plants showed internal structure formation, compared to 17.40 ± 7.00%, and 1.01 ± 1.75% in TaBCAT1-B^R366-^, and TaBCAT1-A^Q50*^ single mutant plants respectively and 1.25 ± 2.80% in double mutant plants (Figure 5C). WT plants had clear hyphal development at 4 dpi (Figure 5E), whereas there was no hyphal growth in the double mutant line at that stage (Figure 5F). By 6 dpi, WT plants showed large, intricate fungal structures (Figure 5G) with a mean area of 0.073 ± 0.026 mm^2^ (Figure 5D). TaBCAT1-B^R366-^ plants showed similar hyphal development to WT plants, with an area of 0.063 ± 0.008 mm^2^. Although hyphal development was detected in TaBCAT1-A^Q50*^ single mutant plants, the structures were significantly smaller (0.0022 ± 0.0015 mm^2^; Figure 5D). Sporadic hyphal development was observed in double mutant plants at 6 dpi, with the area of infection hyphae similar to that of the single mutant lines (0.0044 ± 0.0036 mm^2^). In addition, the double mutant lines showed a unique phenotype at 6 dpi where germinating spores reached stomata and entered the underlying sub-stomatal space, but further fungal growth was absent (Figure 5H). These data indicate that the fungus was inhibited during initial penetration of the TaBCAT1-A^Q50*^ TaBCAT1-B^R366-^ mutant, severely reducing the development of intracellular infection structures.

### *TaBCAT1* mutant lines have constitutively high levels of SA and *PR* gene expression

**Figure 6:**
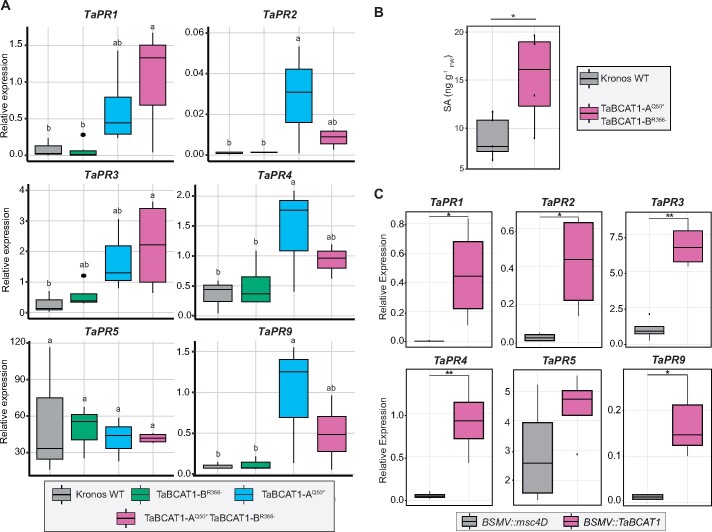
*PR* gene expression and free SA levels are enhanced following disruption of *TaBCAT1* in the absence of infection A. The relative expression of five of the six *PR* genes tested was significantly upregulated in TaBCAT1-A^Q50*^, TaBCAT1-B^R366-^, and/or TaBCAT1-A^Q50*^ TaBCAT1-B^R366-^ disruption mutants in the absence of rust infection. Letters indicate significant differences determined using Duncan’s multi range test (*p *<* *0.05). Kronos WT *n *=* *3, TaBCAT1-A^Q50*^n =* *3, TaBCAT1-B^R366-^*n *=* *3-4, TaBCAT1-A^Q50*^ TaBCAT1-B^R366-^*n *=* *4. B, This was accompanied by a two-fold increase in free SA levels in the TaBCAT1-A^Q50*^ TaBCAT1-B^R366-^ disruption mutant line in the absence of rust infection. Kronos WT *n *=* *5, TaBCAT1-A^Q50*^ TaBCAT1-B^R366-^*n *=* *4. C, The levels of *TaPR1*, *TaPR2*, *TaPR3*, *TaPR4*, and *TaPR9* expression were also significantly higher in *TaBCAT1-*silenced plants. Expression levels were assessed 9 dpvi using RT-qPCR in plants silenced with *BSMV::TaBCAT1* (*n *=* *4) and compared to the negative control, where *BSMV::msc4D* (*n *=* *4) was utilized as a viral infection control. Asterisks denote statistically significant differences between each pair of conditions (**p <* *0.01, *p <* *0.05; two-tailed *t* test). Bars represent median values, boxes signify the upper (Q3) and lower (Q1) quartiles, and whiskers are located at 1.5 the interquartile range

TaBCAT1 is a putative component of BCAA regulation, and the disruption of BCAA metabolism can lead to increased pathogen resistance via SA activation and upregulation of associated *PR* genes (von Saint Paul et al., 2011). To elucidate whether *TaBCAT1* is involved in SA signaling, we measured the expression of six *PR* genes by RT-qPCR in *TaBCAT1* mutant lines in the absence of rust infection (Supplemental Table 2). *TaPR1* and *TaPR3* showed significantly higher levels of transcript accumulation in the double mutants (TaBCAT1-A^Q50*^ TaBCAT1-B^R366-^) compared to WT plants and the single TaBCAT1-B^R366-^ mutant (Figure 6A). The relative expression levels of *TaPR1* and *TaPR3* in TaBCAT1-A^Q50*^ were not significantly different from WT. Transcripts for *TaPR2*, *TaPR4* and *TaPR9* were significantly higher in TaBCAT1-A^Q50*^, whereas the double mutant line TaBCAT1-A^Q50*^ TaBCAT1-B^R366-^ had intermediate levels. *TaPR5* was the only tested *PR* gene with unaltered transcript accumulation in all mutant lines (Figure 6A). Additionally, the double mutant lines contained two-fold more free SA; TaBCAT1-A^Q50*^ TaBCAT1-B^R366^ lines had a mean SA level of 15.20 ± 4.98 ng $g_{\text{FW}}^{-1}$ in comparison to 8.99 ± 2.17 ng $g_{\text{FW}}^{-1}$ in WT plants in the absence of rust infection (Figure 6B).

To evaluate whether the constitutive upregulation of *PR* gene expression in *TaBCAT1* tetraploid TILLING lines was conserved in hexaploid wheat, we measured *PR* gene expression in uninfected plants following virus-induced gene silencing (VIGS). Hexaploid wheat *cv.* Vuka was silenced with the barley stripe mosaic virus (BSMV; Lee et al., 2015) expressing a 251-bp fragment of the *TaBCAT1* gene (Supplemental Figure 10A). By 25 days post-viral inoculation (dpvi), *BSMV::TaBCAT1* plants showed a 93.14-, 14.41-, and 172.43-fold decrease in expression of the A, B, and D genome *TaBCAT1* homoeologs, respectively (Supplemental Figure 10B). *TaPR1*, *TaPR2*, *TaPR3*, *TaPR4*, and *TaPR9* transcript accumulation was significantly higher in *TaBCAT1-*silenced plants in the absence of rust infection at 9 dpvi compared to control *BSMV:msc4D* plants (Figure 6C). *TaPR5* was the only *PR* gene for which expression was only slightly higher in *TaBCAT1*-VIGS plants, in agreement with expression levels observed in the TILLING tetraploid lines. Plants inoculated with the *BSMV::TaBCAT1* construct were also infected independently with *Pst* isolate 13/14 or *Pgt* isolate UK-01. However, silencing was suppressed after *Pst* or *Pgt* inoculation and *TaBCAT1* expression returned to WT levels (Supplemental Figure 10C). Overall, this analysis indicated that the constitutive activation of *PR* genes in *TaBCAT1* TILLING lines can also be observed in hexaploid *TaBCAT1-*silenced plants. The increased levels of SA in *TaBCAT1* mutant lines provide strong evidence for constitutive activation of the defense response, which might protect these plants from pathogens.

### TaBCAT1 contributes to BCAA homeostasis, likely in the mitochondria

**Figure 7:**
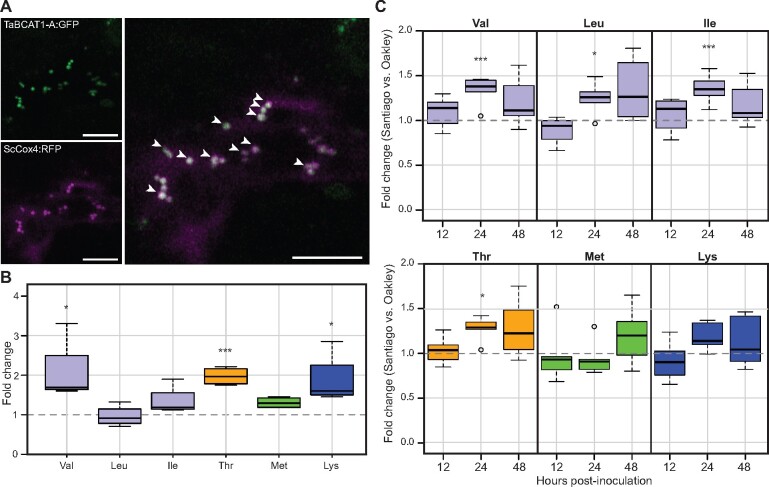
TaBCAT1 localizes to the mitochondria where it may regulate BCAA levels that correspond to *Pst* susceptibility during infection. A, TaBCAT1-A co-localized with the mitochondrial marker ScCOX4. TaBCAT1:GFP and ScCOX4:RFP were transiently co-expressed in *N. benthamiana* and images captured 2-day post-infiltration. White arrowheads highlight overlapping GFP and RFP signals in mitochondria. Images are representative of >10 images captured, which all displayed co-localization of TaBCAT1:GFP and ScCOX4:RFP. Left panels, individual TaBCAT1:GFP (top) and ScCOX4:RFP (bottom) localization patterns, right panel, TaBCAT1:GFP and ScCOX4:RFP merged image illustrating co-localization. Scale bar represents 10 µm. B, Several BCAAs and Asp-derived amino acids were enhanced in TaBCAT1-A^Q50*^ TaBCAT1-B^R366-^ (*n *=* *4) compared to Kronos wild type (*n *=* *5). Val, Thr, and Lys displayed a significant increase in the double mutant. C, The level of the BCAAs Val, Leu, and Ile and the Asp-derived amino acid Thr were significantly increased at 24 hpi in the resistant variety Santiago (*n *=* *5) during *Pst* infection (isolate F22) when compared to the susceptible variety Oakley (*n *=* *5). The level of the BCAAs and Asp-derived amino acids were assessed at 12, 24, and 48 hpi. Asterisks denote statistically significant differences (***p <* *0.001, *p <* *0.05; two-tailed *t* test). Bars represent median values, boxes signify the upper (Q3) and lower (Q1) quartiles, and whiskers are located at 1.5 the interquartile range

To infer the potential subcellular location of TaBCAT1, we analyzed the three homoeologs for predicted N-terminal sorting signals. This analysis indicated potential mitochondrial localization for TaBCAT1-A, TaBCAT1-B, and TaBCAT1-D, with iPSORT (Nakai and Horton, 1999) identifying a mitochondrial targeting peptide in the first 30 amino acids and MitoProt (Claros, 1995) and TargetP (Emanuelsson et al., 2000) reporting a high probability of mitochondria localization (Supplemental Table 3 and Supplemental Figure 7). To further assess the subcellular localization of TaBCAT1, we fused GFP to the C-terminus of TaBCAT1-A and transiently co-expressed TaBCAT1:GFP alongside the mitochondrial marker ScCOX4:RFP in *N. benthamiana* (Petre et al., 2015). The intracellular localization of TaBCAT1:GFP assessed using confocal microscopy indicated clear co-localization with ScCOX4:RFP, further supporting the mitochondrial subcellular location of TaBCAT1 (Figure 7A).

We hypothesized that the localization of TaBCAT1 to the mitochondria may indicate an involvement in BCAA degradation in a similar manner to *A. thaliana* AtBCAT1 (Schuster and Binder, 2005). If this were the case, disrupting the gene could cause BCAA accumulation. Accordingly, we measured the levels of free amino acids including BCAAs (Leu, Ile, and Val) and Asp-derived amino acids Thr, Met, and Lys in TaBCAT1-A^Q50*^ TaBCAT1-B^R366-^. Amino acid levels were generally moderately higher in the double mutant line when compared to the WT, including all BCAAs and Asp-derived amino acids except Leu (Figure 7B;Supplemental Figure 11A). Regarding BCAAs, Val and Ile showed higher amounts when compared to the WT (2.07- and 1.35-fold, respectively) with Val showing a statistically significant increase. Leu was present at WT levels. For other Asp-derived amino acids, Thr was 2-fold higher, Met 1.3-fold higher, and Lys 1.9-fold higher in TaBCAT1-A^Q50*^ TaBCAT1-B^R366-^. In summary, TaBCAT1 putatively localizes to the mitochondria, where it appears to regulate Asp-derived amino acid levels.

The enhanced levels of BCAAs Val and Ile and Asp-derived amino acids observed in the TaBCAT1-A^Q50*^ TaBCAT1-B^R366-^ mutant corresponded with an increase in SA levels and decrease in *Pst* and *Pgt* susceptibility potentially via constitutive activation of the defense response. To determine if the elevation in BCAAs and Asp-derived amino acids levels could be broadly linked to *Pst* resistance responses, we compared the amino acid levels in the most resistant variety Santiago to those of the susceptible variety Oakley early during infection with *Pst* isolate F22 (12, 24, and 48 hpi). At 12 hpi the levels of all amino acids were comparable between varieties (Figure 7C;Supplemental Figure 11B). However, at 24 hpi the BCAAs Val, Leu, and Ile and the Asp-derived amino acid Thr showed significantly higher amounts in Santiago compared to Oakley during *Pst* infection (1.33-, 1.25-, 1.35-, and 1.27-fold, respectively), whereas the levels of the Asp-derived amino acids Met and Lys were alike. By 48 hpi, the levels of all BCAAs and Asp-derived amino acids began to return to those seen at 12 hpi, with a high degree of variation between replicates likely due to non-synchronous progression of the infection process. This analysis illustrates that the levels of BCAAs and Thr tightly associate with the degree of resistance of the wheat variety during *Pst* infection.

### Exogenous application of ILA perturbs *Pst* infection

**Figure 8:**
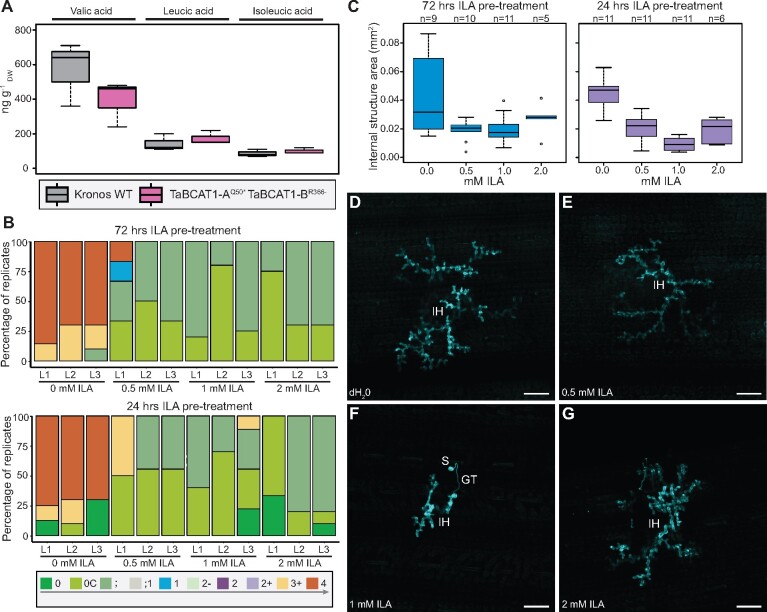
Levels of the three BCAA-related 2-hydroxyacids are unchanged in the *TaBCAT1* disruption mutant, but exogenous application of ILA severely perturbs *Pst* infection. A, Levels of the three BCAA-related 2-hydroxyacids (ILA, VA, and LA) were determined in the *TaBCAT1* disruption mutant and Kronos wild-type plants in the absence of rust infection. Leaves from three independent plants were assessed in each case. B, Exogenous application of ILA 72 or 24 h prior to *Pst* infection dramatically reduced disease progression. Oakley plants were pre-treated with ILA at 0.5, 1, and 2 mM or with dH_2_0 (0-mM ILA) 72 or 24 h prior to inoculation with *Pst* isolate 13/14 and infection types recorded 15-day post-infection using the 0–4 scale (McIntosh et al., 1995). Values represent an average from 10 plants per treatment, with the first (L1), second (L2), and third (L3) leaves analyzed. C, Histological studies using a fungus-specific fluorescent dye revealed that where internal fungal structures formed, their extension was only moderately reduced following ILA treatment at 0.5, 1, or 2 mM prior to *Pst* infection. D–G, Images illustrate the moderate reduction in *Pst* internal fungal structures when plants were treated with ILA 24 h prior to *Pst* infection. Scale bar represents 100 µm. S, urediniospore; GT, germ tube; SSV, sub-stomatal vesicle; IH, invasive hyphae. For the box plots, the bars represent median values, boxes signify the upper (Q3) and lower (Q1) quartiles, and whiskers are located at 1.5 the interquartile range

Levels of BCAAs and BCAA-related 2-hydroxyacid ILA are associated in *Arabidopsis*. Furthermore, exogenous application of ILA can enhance SA-mediated defense gene expression and resistance to *P. syringae* (von Saint Paul et al., 2011). Therefore, we analyzed the levels of the three BCAA-related 2-hydroxyacids including ILA in the TaBCAT1-A^Q50*^ TaBCAT1-B^R366-^ double mutant and WT plants. Similar levels of the three BCAA-related 2-hydroxyacids [ILA, valic acid (VA), and leucic acid (LA)] were observed between the double mutant and WT plants (Figure 8A). However, the levels of the three compounds varied, with ILA being the least abundant (WT: 86.67 ± 20.82 ng $g_{\text{DW}}^{-1}$; TaBCAT1-A^Q50*^ TaBCAT1-B^R366-^: 100 ± 17.32 ng $g_{\text{DW}}^{-1}$). These levels were considerably lower than those previously reported for ILA in other species including the monocot *H. vulgare* (∼550 ng $g_{\text{DW}}^{-1}$) and are at the lower limit of detection with the current assay (Maksym et al., 2018). Thus, with the current sensitivity of the assay, the disruption of *TaBCAT1* was not shown to associate with an alteration in the levels of the three BCAA-related 2-hydroxyacids.

To determine if ILA application can activate defense against biotrophic pathogens in wheat, we exogenously applied ILA to the wheat variety Oakley at 72 and 24 h prior to infection with *Pst* isolate 13/14. Histological studies were conducted at 6 dpi with a fungus-specific fluorescent dye (Koch and Pimsler, 1987) and ITs assessed 16 dpi following the 0–4 scale (McIntosh et al., 1995). This analysis revealed a dramatic reduction in *Pst* disease progression (Figure 8B). Where limited internal hyphae did develop following ILA treatment, their extension was only moderately reduced when compared to the dH_2_0 control (0-mM ILA). This reduction in extension of invasive hyphae was more pronounced for those plants pre-treated with ILA at 24 h prior to *Pst* infection (Figure 8, C–G). Thus, similar to reports in Arabidopsis, ILA application in wheat enhances resistance to biotrophic pathogens such as *Pst*.

## Discussion

Advances in transcriptome sequencing and improved genomic resources for many crop species offer an unprecedented opportunity to identify genetic regions contributing to plant immune responses and pathogen susceptibility. Moreover, exploring the wide-ranging susceptibility displayed across plant cultivars in field conditions provides exceptional sensitivity for identifying novel mechanisms controlling plant disease progression. Herein, we demonstrate the suitability of field-based RNA-seq analysis to identify defense components by analyzing wheat varieties with different degrees of susceptibility. We sourced samples of the same wheat variety from different environmental conditions and/or developmental stages to identify genes linked to *Pst* susceptibility, as the main commonality between samples was their response to *Pst* infection. This method led to the successful identification of a gene (*TaBCAT1*) that plays an essential role as a positive regulator of susceptibility during *Pst* and *Pgt* disease progression. To our knowledge, this represents the first report of a role for BCATs in disease susceptibility and illustrates the value of the approach described herein. This approach could provide a novel alternative to traditional, labor-intensive and time-consuming methods to identify genes that condition susceptibility, most of which rely on screening mutant populations or identifying the intrinsic resistance in wild relatives or natural populations.

**Figure 9:**
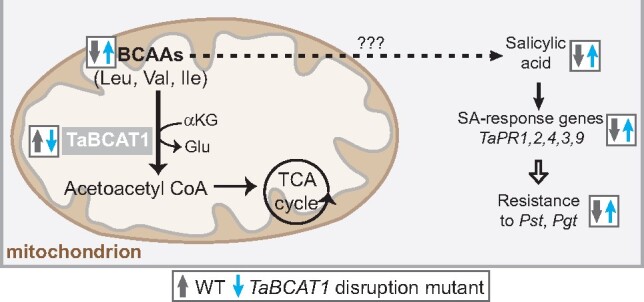
Model illustrating the role of TaBCAT1 in the defense response. Disruption of *TaBCAT1* (downward blue arrow) leads to an elevation in BCAA levels, free SA accumulation and enhanced expression of pathogenicity-related genes (*TaPR1, 2, 4, 3*, and *9*), stimulating an increase in resistance to *Pst* and *Pgt* infection (upward blue arrows). The connection between elevated BCAAs and SA is currently unknown (dotted arrow). In contrast, wild type (WT) expression of *TaBCAT1* (upward gray arrow) leads to lower levels of BCAAs, SA, *PR* gene expression, and resistance to *Pst* (downward gray arrows)

Our analysis revealed *TaBCAT1* as essential for wheat rust susceptibility, with a likely role early in infection via BCAA regulation, which is crucial for SA-dependent SAR activation in wheat (Figure 9). The activation of SAR through SA accumulation can lead to defense priming, where plant defenses are preconditioned and thereby more rapidly respond to subsequent infection (Jung et al., 2009). The TaBCAT1-A^Q50*^ TaBCAT1-B^R366-^ double mutant had two-fold more constitutive free SA, indicating potential “priming” of defense machinery. Such constitutive activation of the immune system would account for its increased *Pst* and *Pgt* resistance, although how changes in BCAA metabolism trigger SA-dependent defense responses is currently unknown. We also identified a general association between the levels of BCAAs (Val, Leu, and Ile) and the Asp-derived amino acid Thr and resistance to *Pst* infection in wheat. It is possible that increasing the available levels of the Asp-derived amino acid Ile could lead to conversion to ILA, which activates SA-dependent SAR and *PR* gene expression (von Saint Paul et al., 2011). In *A. thaliana*, the levels of the BCAA-related compound ILA correlate with the expression of a glucosyltransferase (UGT76B1) that is induced in response to stress (von Saint Paul et al., 2011). Similar to *TaBCAT1* mutant lines, *ugt76b1* mutants exhibit activation of defense-related genes and increased levels of free SA. However, the levels of ILA in the *TaBCAT1* double mutant were comparable to those of the WT, indicating that the activation of SA in this instance was likely independent of ILA accumulation. Taken together, our data establish a link between the absence of *TaBCAT1* and an increase in BCAA levels leading to SA induction. We hypothesize that it could be advantageous for the pathogen to elevate expression of *TaBCAT1* early during infection to suppress activation of SA-mediated defense responses.

An elevation in SA levels during pathogen infection has been widely shown to activate *PR* gene expression (Gaffney et al., 1993). *TaBCAT1* disruption also led to constitutive expression of all *PR* genes tested except *TaPR5*. In wheat, *PR* gene expression is cultivar and pathogen specific and induced in a complex manner by SA and JA (Jayaraj et al., 2004; Zhang et al., 2018). For instance, in the wheat cultivar Alpowa exogenous SA treatment triggers expression of *TaPR1*, *TaPR2*, *TaPR3*, *TaPR4*, and *TaPR5*, while JA does not activate these genes. In contrast, in the wheat cultivar Chinese Spring, SA and JA treatments trigger expression of *TaPR1*, *TaPR3*, and *TaPR10* (Zhang et al., 2018). Here we showed that *PR* expression was significantly elevated in the absence of infection in the double and single TaBCAT1-A^Q50*^ mutant lines, while the single TaBCAT1-B^R366-^ mutant exhibited WT-like expression levels for all *PR* genes tested. These observations suggest a stronger effect of the mutation in *TaBCAT1-A* and/or limited functionality of *TaBCAT1-B* in Kronos wheat. Additionally, constitutive *PR* activation was observed in the hexaploid wheat variety Vuka during *BSMV*-induced *TaBCAT1* silencing. Together, these results support a role for *TaBCAT1* in suppressing SA-mediated resistance.

TaBCAT1 shares sequence and structural similarity with members of the *A. thaliana* AtBCAT family. AtBCAT1 localizes to the mitochondria and is involved in BCAA degradation (Schuster and Binder, 2005). Similarly, TaBCAT1 contains a mitochondrial targeting signal and localized to the mitochondria when transiently expressed in *N. benthamiana*. The BCAA accumulation observed in *TaBCAT1* mutants also supports a role for TaBCAT1 in BCAA degradation. A strong connection between Asp-derived amino acid catabolism and the tricarboxylic acid (TCA) cycle has been previously suggested; BCAA degradation initiated by BCAT has been proposed to lead to oxoacid generation and its subsequent transformation to metabolites for the TCA cycle (Galili, 2011). Accordingly, degradation of BCAA could be crucial during stress conditions to provide alternative carbon sources to obtain energy via the TCA cycle. This could be exploited by the pathogen to sequester carbon sources from the host plant and would align with the increase in *TaBCAT1* expression and lower levels of BCAAs observed in *Pst* compatible interactions.

The decrease in susceptibility and enhanced levels of SA displayed by *TaBCAT1* disruption mutants suggests that TaBCAT1 may act as a suppressor of basal defense responses. Genes linked to disease susceptibility and with roles in metabolism, including amino acid metabolism, have been reported previously. For example, *DMR* genes encode a homoserine kinase that converts homoserine to homoserine‐4‐phosphate, a step in the Asp-derived amino acid pathway (Van Damme et al., 2005). *Dmr* mutants also display constitutive expression of *PR1*. TaBCAT1 is required for pathogen colonization and may act in a similar manner in wheat, catalyzing a different step in the Asp-derived amino acid pathway. Genes essential for pathogen ingress often confer broad-spectrum resistance to different pathogen races and species. Indeed, *TaBCAT1* Kronos disruption mutants conferred seedling resistance to both *Pst* and *Pgt*. Further work is required to determine whether this response is conserved in other wheat varieties, and in response to alternative *Pst* and *Pgt* races and/or other pathogens.

The identification and exploration of *TaBCAT1* provides insight into SA-mediated defense responses in wheat. Together with the general association we uncovered between the levels of BCAAs and resistance to *Pst* infection, our results highlight the role of BCAA metabolism in the defense response. Furthermore, given the crucial role of *TaBCAT1* in pathogen ingress, this gene would be an interesting target for manipulation to provide a new source of resistance to *Pst* and *Pgt* infection.

## Materials and methods

### RNA-seq of *Pst*-infected wheat samples

A total of 156 *Pst*-infected bread wheat (*T. aestivum*) leaf samples were collected between 2013 and 2016 and stored to maintain nucleic acid integrity in RNAlater solution (Thermo Fisher Scientific, Paisley, UK), of which 130 were previously processed for RNA-seq analysis (Hubbard et al., 2015; Bueno-Sancho et al., 2017; Supplemental Data Set 1). The remaining 26 samples were subjected to RNA extraction using the Qiagen RNeasy Mini Kit according to the manufacturer’s instructions (Qiagen, UK), and cDNA libraries were prepared using an Illumina TruSeq RNA Sample Preparation Kit (Illumina, USA). Libraries were sequenced on the Illumina HiSeq 2500 machine (Earlham Institute, UK and GENEWIZ, Germany), and adapter and barcode trimming and quality filtering were performed on the resulting 101-bp pair-end reads using the BBDuk tool in BBtools version 37.68 (Bushnell, 2019). Reads were aligned independently to the *Pst* reference genome (isolate PST-130; Cantu et al., 2011) and wheat reference genome (IWGSC RefSeq v1.0; International Wheat Genome Sequencing et al., 2018) using TopHat (Trapnell et al., 2012) and STAR version 2.5a (Dobin et al., 2013), respectively. SNP sites were determined using SAMtools version 0.1.19 (Li et al., 2009), considering only sites with a minimum depth of coverage of 20x. Next, synthetic gene sets for each wheat sample were generated that incorporated sites that met a minimum of 20x depth of coverage for those that differed from the reference sequence and 2x coverage if they were identical, using the method described previously (Hubbard et al., 2015). A subset of 96 *Pst*-infected wheat leaf samples were selected and these synthetic gene sets used for phylogenetic analyses, performed with a maximum-likelihood approach using RaxML 8.0.20 (Stamatakis, 2006). Phylogenetic trees were visualized in Dendroscope version 3.5.10 (Huson et al., 2007). Wheat varieties were confirmed through sequence analysis at a set of 1,831 genetically mapped SNP positions (Wang et al., 2014) following methods described previously (Hubbard et al., 2015). Wheat varieties with a minimum of 12 SNP sites with ≥20x coverage were used for varietal analysis.

### Differential gene expression analysis

Transcript abundance was assessed for each of the 21 *Pst*-infected Oakley, Solstice and Santiago samples using pseudoalignments to the wheat reference transcriptome (IWGSC RefSeq v1.0; International Wheat Genome Sequencing et al., 2018) generated by kallisto version 0.43.0 (Bray et al., 2016). Following pseudoalignment, additional normalization was performed by importing raw read count data into R using tximport version 1.8.0 and subsequently the R package RUVseq 1.16.1 (Risso et al., 2014). Principal component analysis was performed and RLE values assessed following normalization using three separate methods: (1) raw read count data normalized using an approach known as RUV using control genes; (2) counts normalized by RUV using residuals; and (3) counts normalized by RUV using replicate samples. The latter method was selected as the most effective in reducing RLE distribution. The resulting normalized transcript per million (t.p.m.) data were used to generate co-expression clusters using Clust (Abu-Jamous and Kelly, 2018). t.p.m. values were log_2_-normalized and the python library goatools used to functionally annotate genes in each of the co-expression clusters using GO terms associated with the IWGSC RefSeq v1.1 transcripts (International Wheat Genome Sequencing et al., 2018). GO term processes were assessed for Enrichment using Fisher's exact test and GO terms considered enriched in one cluster at *p* < 0.0001. Enriched GO terms at levels 2 and 3 were selected for each co-expression cluster and visualized using ggplot2 (Wickham, 2016).

### RT-qPCR analysis of gene expression in *Pst*-infected wheat samples

Two-week-old seedlings of the hexaploid wheat (*T. aestivum* L.) varieties Oakley, Solstice and Santiago were infected independently with urediniospores of *Pst* isolates 13/14 or F22 (Hubbard et al., 2015). Plants were pre-germinated and sown in cell trays (60 plants per variety) and grown in controlled-environment long day conditions with 16 h of light at approximately 250 μmol m^−2^ s^−1^ and 8 h of dark under a 19/14°C cycle. The main lighting used bulbs manufactured by Philips (UK; model CDM-TMW 315w/930) and was supplemented with Valoya (Finland) NS1 LEDs. *Pst* urediniospores were heat-activated at 40°C for 5 min and then resuspended in Novec 7100 (Sigma-Aldrich, USA; 1 mg·mL^–1^) to facilitate spray inoculation. For control experiments, a mock spray inoculation with Novec 7100 was performed. Following inoculation, plants were initially maintained in darkness at 10°C, with high relative humidity for 24 h before being transferred to the aforementioned controlled environment conditions. ITs were determined 12 dpi in approximately six plants per variety following the 0–4 scale (McIntosh et al., 1995). Leaf samples were collected at 12 hpi and 2, 5, 9, and 11 dpi. Two independent leaves from the same plant were pooled and three biological replicates (different plants) were collected per time point, condition and wheat variety. Samples were snap frozen in liquid nitrogen and stored at −80°C to preserve RNA integrity.

RNA was extracted from a total of 80–100 mg of leaf material using the Qiagen Plant RNeasy Mini Kit (Qiagen, UK) according to the manufacturer’s instructions. DNA was removed from RNA samples using the TURBO DNA-free Kit (Ambion, UK), and the quantity and quality of RNA assessed using the Agilent 2100 Bioanalyzer (Agilent Technologies, UK). cDNA was synthesized using SuperScript™ II Reverse Transcriptase (Invitrogen, USA) and random hexamers and Oligo(dT) primers according to the manufacturer’s instructions. RT-qPCR was performed using LightCycler 480 SYBR Green I Master Mix (Roche, Switzerland) following the manufacturer’s instructions with each primer at a final concentration of 0.25 µM. Primer sequences are provided in Supplemental Table 2. *TaBCAT1* expression was compared between *Pst*-infected and mock-inoculated plants for each time point and variety.

### Annotation and cloning of *TaBCAT1* for subcellular localization

The expression levels of 37 transcripts annotated as involved in BCAA biosynthesis were assessed during fungal infection in publicly available wheat RNA-seq data using the wheat expression browser (Borrill et al., 2016; Figure 3). The predicted subcellular location of the three homoeologous copies of *TaBCAT1* (TraesCS4A02G059800.1, TraesCS4B02G235400.3, TraesCS4D02G236800.1) was assessed using iPSORT (Nakai and Horton, 1999), MitoProt (Claros, 1995), and TargetP (Emanuelsson et al., 2000) using default parameters (Supplemental Table 3). Sequence alignments were performed using Clustal omega (Sievers and Higgins, 2014) and visualized in Jalview (Clamp et al., 2004). Sequence similarity of TaBCAT1 to the *A. thaliana* AtBCAT family was assessed using BLASTp (Altschul et al., 1990).

For subcellular localization of TaBCAT1, TaBCAT1-A was PCR amplified from cDNA of wheat variety Santiago using the primer sequences 5TaBCAT1-A (ACGTGCGTGCATGGCT) and 3TaBCAT1-A (GCCCACGCTGTTATCCTAC). Next, the initial TaBCAT1-A amplicon was used as template in a second PCR reaction to add the Gateway compatible CACC overhang using the primer sequences 5TaBCAT1-AENTR (CACCATGGCTGTGCTGTCGTCTGCGA) and 3TaBCAT1-AENTR (ATCAACCGCGACCGTCCATCCCT). The resulting TaBCAT1-A amplicon was cloned into the entry vector pENTR/D-TOPO (Invitrogen, USA) and resulting clone confirmed by sequencing. The entry clone insert was then introduced into pK7WGF2 (Karimi et al., 2002) by Gateway LR recombination (Invitrogen, USA). The resulting C-terminal GFP fusion construct (TaBCAT1:GFP) was transformed into *Agrobacterium tumefaciens* strain GV3101 and transiently expressed in *Nicotiana benthamiana* following methods described previously (Bos et al., 2006).

### Evaluation of *TaBCAT1* TILLING wheat lines

Tetraploid wheat TILLING lines (*cv.* Kronos; Uauy et al., 2009; Krasileva et al., 2017) with disruption mutations in *TaBCAT1* were selected. For *TaBCAT1-A* (TraesCS4A02G059800), we selected a TILLING mutant line (Kronos2898) with a C-to-T transition, which led to a premature stop codon at amino acid 50. For *TaBCAT1-B* (TraesCS4B02G235400), we selected a TILLING mutant line (Kronos860) with a G-to-A transition that was predicted to cause a mis-splicing variant, leading to protein truncation at amino acid 362. Leaf fragments were used for DNA extraction (Pallotta et al., 2003) of TILLING mutant plants, and genotypic analysis was carried out using Kompetitive allele-specific PCR (LGC Genomics, UK) as described previously (Ramirez-Gonzalez et al., 2015) using subgenome-specific primer sequences (Supplemental Table 4). Heterozygous single mutant lines were self-pollinated to develop homozygous mutant lines. Next, homozygous single mutant lines (TaBCAT1-A^Q50*^ and TaBCAT1-B^R366-^) were crossed to obtain the double mutant line TaBCAT1-A^Q50*^ TaBCAT1-B^R366-^. Genotypic analysis of F_2_ progeny was used to identify double homozygous mutant plants. TaBCAT1-A^Q50*^, TaBCAT1-B^R366-^, and TaBCAT1-A^Q50*^ TaBCAT1-B^R366-^ mutant lines and Kronos WT plants were spray inoculated with rust urediniospores from the *Pst* isolate 13/14 (Hubbard et al., 2015) and *Pgt* isolate UK-01 (Lewis et al., 2018) as described above. Disease symptoms were evaluated 20 days after *Pst* infection and 16 days post *Pgt* infection. The percentage of infection in independent leaves was measured using K-PIE (Bueno-Sancho et al., 2019).

### VIGS using the BSMV

A 251-bp fragment of *TaBCAT1* was cloned into the BSMV vector pCa-γbLIC (Yuan et al., 2011) via ligation independent cloning as described previously (Lee et al., 2015). The VIGS target sequence was designed to target the three homoeologous copies of the gene simultaneously and was amplified using the primer sequences VIGS-F (GGCCTCCGAGCTCTACTC) and VIGS-R (GGGGCTGAGCTCGATGTTG). *BSMV::TaPDS* was used as a positive control and *BSMV::msc4D* as a viral infection control (Lee et al., 2015). All constructs were independently transformed into *A. tumefaciens* strain GV3101 and transiently expressed in *N. benthamiana* according to methods described previously (Bos et al., 2006). Three to five days post agro-infiltration, sap was harvested from *N. benthamiana* and used to mechanically inoculate four 9-day-old wheat seedlings (*cv.* Vuka) per construct. After 9 days, seedlings were inoculated with rust urediniospores as described above. Three biological replicates (independent experiments) were performed. Leaves were harvested 25 days post-viral inoculation and snap frozen in liquid nitrogen. Silencing was assessed using RT-qPCR with primers outside of the VIGS target region (Supplemental Table 2).

### Quantification of free SA and amino acid contents

Five 11-day-old Kronos WT seedlings, four TaBCAT1-A^Q50*^ TaBCAT1-B^R366-^ seedlings, and five *Pst*-infected Oakley and Santiago seedlings were harvested by flash-freezing in liquid nitrogen. A total of 100–300 mg of plant tissue per sample was lyophilized for 48 h. Soluble amino acids were extracted and quantified from all samples and free salicylate levels from Kronos WT and the TaBCAT1-A^Q50*^ TaBCAT1-B^R366-^ mutant plants as described previously (Winter et al., 1992) with minor modifications. In short, a 60-µL solution containing 20-mM HEPES (pH 7.0), 5-mM EDTA and 10-mM NaF, and 250 µL of chloroform/methanol (1.5/3.5, v/v) were added to the lyophilized plant material. Next, 1 µL of internal standard salicylate-d4 (100 µM) was added to samples used for free salicylate extraction and samples homogenized using the TissueLyser II (Qiagen, UK) with one Tungsten 3-mm bead per tube and then incubated on ice for 30 min. The homogenate was centrifuged at 15,871 g for 10 min and the aqueous phase collected, and the dried residue washed in 300 µL water. Salicylate was measured on an Acquity UPLC attached to a TQS tandem mass spectrometer (Waters, UK). Separation of metabolites in the sample extract was performed on a 50 mm × 2.1 mm, 2.7 μm Kinetex EVO-C18 column (Phenomenex, USA) using a two-minute gradient of 10 to 80% acetonitrile versus 0.1% formic acid in water, run at 0.6 mL min^−1^ and 35°C. Salicylate and the internal standard were monitored by negative mode electrospray using the transitions 137→93 or 141→97. The levels of free amino acids were also measured using this instrument. Samples were filtered using microcentrifuge tube filters and diluted to 1:100 in water. A total of 10 µL of each sample was then derivatized using a Waters’ AccQ tag kit according to the manufacturer’s instructions (Waters, UK) and 2 µL used for injection. Separation of amino acids in the sample extract was on a 100 mm × 2.1 mm, 2.7-μm Kinetex XB-C18 column (Phenomenex, USA) using a 14.5 min gradient of 1%–20% acetonitrile versus 0.1% formic acid in water, run at 0.58 mL·min^−1^ and 25°C. The mass transitions, in positive mode, were 290→170.58 (Thr), 320→171.05 (Met), 244→170.58 (Lys), 246→171.34 (Gly), 260→170.58 (Ala), 276→171.15 (Ser), 286→170.58 (Pro), 304→170.58 (Asp), 317→170.67 (Gln), 326→170.86 (His), 345→170.86 (Arg), 352→170.67 (Tyr), 336→170.58 (Phe).

### Quantification of VA, LA, and ILA

Three 14-day-old seedlings of TaBCAT1-A^Q50*^ TaBCAT1-B^R366-^ and Kronos WT were harvested by flash-freezing in liquid nitrogen. Approximately 20 mg of lyophilized, ground plant tissue per sample was extracted as described previously (Maksym et al., 2018) with minor adjustments. A total of 1 mL of 70%/30% methanol/H_2_0 (v/v) containing 0.625 mg·L^-1^ of 4-nitrophenol (internal standard) was precooled to 4°C and added to each sample. Samples were sonicated at 4°C for 15 min, before being shaken for 60 min and sonicated for a further 15 min at 4°C. The subsequent extract was centrifuged for 15 min and 700 µL transferred to a glass vial and evaporated using a speedvac (Genevac, UK). A total of 50 µL of BSTFA (N,O-Bis(trimethylsilyl)trifluoroacetamide, Sigma-Aldrich, USA) containing 1% TMCS (Trimethylchlorosilane, Sigma-Aldrich, USA) was added and the sample derivatized at 60°C for 120 min. Standard solutions for VA, LA, and ILA were prepared in 70%/30% methanol/H_2_0 (v/v) containing 0.625 mg·L^−1^ of 4-nitrophenol (internal standard) at 0.1, 0.05, 0.025, 0.01, and 0.005 mg·L^−1^. A total of 700 µL of each solution was evaporated and derivatized in the same way as the sample extracts.

Qualitative and quantitative analysis was performed by GC-MS using an Agilent 7890B GC coupled to an Agilent 5977A MSD. The column used was a Zebron Inferno ZB-5HT 30m + 5m guard 0.25 mm × 0.1 µm (Phenomenex, USA) with helium carrier gas at 0.6 mL·min^−1^. Samples were injected (1 µL) at 250°C using split injection with a split ratio of 10:1. The oven was initially held at 50°C for 1 min then ramped at 15°C·min^−1^ to 110°C, held for 5 min, ramped at 100°C·min^-1^ to 300°C and held for 5 min. The MS was operated in SCAN and SIM mode operating over a m/z range of 30–300 in scan mode and monitoring masses 103,133,117,145,147,159,196 in SIM mode. Target ions were 145 (VA), 159 (LA, ILA), and 196 (Internal standard), with the other ions used as qualifier ions.

### Confocal microscopy

Leaf samples of TaBCAT1-A^Q50*^, TaBCAT1-B^R366-^, TaBCAT1-A^Q50*^ TaBCAT1-B^R366-^, and Kronos WT infected with *Pst* isolate 13/14 were collected at 4 and 6 dpi and stained using Uvitex-2B (Generon, UK; Moldenhauer et al., 2006). Specimens were mounted in water on microscope slides and examined under a Zeiss LSM780 and LSM880 Airyscan confocal microscopes (Zeiss, Germany) using a Plan NeoFluar 10x/NA 0.3 objective and a Plan Apochromat 10x/NA 0.45 objective, respectively, with an excitation wavelength of 405 nm (30 mW diode laser) and emission bandwidth of 410–468 nm. Stacks were recorded on a PMT detector with a 1 Airy unit pinhole and voxel dimensions of 0.83 × 0.83 × 2.43 µm. Seven independent samples of Kronos WT, four samples of single mutant plants (TaBCAT1-A^Q50*^ and TaBCAT1-B^R366-^) and three samples of TaBCAT1-A^Q50*^ TaBCAT1-B^R366-^ were examined by confocal microscopy.

For co-localization experiments of TaBCAT1:GFP with the mitochondrial marker ScCOX4:RFP, approximately 1 cm^2^ patches of *N. benthamiana* leaves were cut and mounted in water. Slides were imaged on a Zeiss LSM880 Airyscan confocal microscope using 488 nm (25-mW Argon Ion laser) and 561 nm (20-mW DPSS laser) excitation with emission bandwidths of 495–550 nm and 570–620 nm, respectively. The SR-option of the Airyscan Fast mode was used with a Plan Apochromat 40x/NA 1.1 water-immersion objective and single planes were taken with pixel dimensions of 54 × 54 nm followed by 2D Airyscan processing with default settings (Zeiss, Germany).

### Exogenous application of ILA

Twenty 2-week-old seedlings of wheat variety Oakley were treated with ILA ([(2S, 3S)-2-hydroxy-3-methylpentanoic acid]; abcr, Germany) at 0.5, 1, or 2 mM (diluted in dH_2_O), alongside a dH_2_O control (0 mM ILA). Plants were initially covered for 1–2 h and then incubated in a glasshouse under long-day conditions (16-h light/8-h dark) and 19/14°C cycle. After 24 or 72 h, 10 plants from each treatment were inoculated with *Pst* isolate 13/14. Leaf samples were collected 6 dpi and stained using Uvitex-2B (Generon, UK; Moldenhauer et al., 2006). Specimens were mounted on microscope slides and examined as detailed above. Fluorescence and confocal microscopy were performed on 6–11 independent leaf fragments per treatment. Macroscopic infection symptoms were assessed 15 dpi following the 0–4 scale (McIntosh et al., 1995).

### Statistical analysis

Duncan’s multiple range test (DMRT) was used to determine significant differences between pairs of means for TaBCAT1-A^Q50*^, TaBCAT1-B^R366-^, TaBCAT1-A^Q50*^ TaBCAT1-B^R366-^ and Kronos WT following infection with *Pst* when analyzing the percentage of germinating spores forming internal structures, area of internal fungal structures formed and leaf infection area following infection with *Pst* or *Pgt*. DMRT was also used to compare *PR* gene expression between TaBCAT1-A^Q50*^, TaBCAT1-B^R366-^, TaBCAT1-A^Q50*^ TaBCAT1-B^R366-^, and Kronos WT. A two-tailed Student’s *t* test was used to evaluate the significance of differences in (1) leaf infection area between TaBCAT1-A^Q50*^, TaBCAT1-B^R366-^, TaBCAT1-A^Q50*^ TaBCAT1-B^R366-^, and Kronos WT, (2) *TaBCAT1* expression between wheat varieties during *Pst* infection (isolates F22 and 13/14), (3) SA levels between TaBCAT1-A^Q50*^ TaBCAT1-B^R366-^ and Kronos WT, (4) *PR* and *TaBCAT1* expression between plants inoculated with the silencing constructs *BSMV::TaBCAT1* and *BSMV::msc4D*, (5) amino acid levels between TaBCAT1-A^Q50*^ TaBCAT1-B^R366-^ and Kronos WT and wheat varieties Santiago and Oakley infected with *Pst* (isolate 13/14), and (6) levels of VA, LA, and ILA between TaBCAT1-A^Q50*^ TaBCAT1-B^R366-^ and Kronos WT. DMRT was conducted in R and *t* tests in R and Excel, with further details of statistical analyses provided in Supplemental Data Set 3.

### Accession numbers

Sequence data from this article can be found in the European Nucleotide Archive (ENA) database under the following accession number: ENA:PRJEB36444. Individual accession numbers for all publicly available sequence data used are provided in Supplemental Data Set 1. All custom computer code has been deposited on github: https://github.com/pilarcormo/Wheat-response-PST.

## Supplemental data

The following materials are available in the online version of this article.

**Supplemental Figure 1.** *Pst*-infected wheat samples from the varieties Oakley, Solstice, and Santiago selected for transcriptome analysis.

**Supplemental Figure 2.** Evaluation of normalization of transcript count data using principal component analysis from *Pst*-infected Oakley, Solstice, and Santiago samples.

**Supplemental Figure 3.** Evaluation of normalization of transcript count data using relative log expression (RLE) values from *Pst*-infected Oakley, Solstice, and Santiago samples.

**Supplemental Figure 4.** Biological processes enriched in one or more of the 12 co-expression clusters.

**Supplemental Figure 5.** Cellular components enriched in one or more of the 12 co-expression clusters.

**Supplemental Figure 6.** Molecular functions enriched in one or more of the 12 co-expression clusters.

**Supplemental Figure 7.** Multiple sequence alignment of the protein sequences of the three *T. aestivum* homeologs of TaBCAT1.

**Supplemental Figure 8.** *TaBCAT1* disruption mutants display a significant reduction in susceptibility to *Pgt*.

**Supplemental Figure 9.** *TaBCAT1* expression early during *Pst* infection is linked to wheat varietal susceptibility.

**Supplemental Figure 10.** Virus-induced gene silencing of *TaBCAT1*.

**Supplemental Figure 11.** Levels of the BCAAs Val and Ile are enhanced in the *TaBCAT1* disruption mutant and of the remaining amino acids, Ser and Asp levels were modulated dependent on the level of *Pst* susceptibility during infection.

**Supplemental Table 1.** Seedling infection assays illustrate that the three wheat varieties Oakley, Solstice and Santiago have different levels of susceptibility to the *Pst* isolates F22 and 13/14.

**Supplemental Table 2.** RT-qPCR primer sequences and efficiencies.

**Supplemental Table 3.** Probability of mitochondrial localization for TaBCAT1 homoeologous proteins.

**Supplemental Table 4.** KASP primers to genotype TILLING lines selected.

**Supplemental Data Set 1.** *Pst*-infected field-collected wheat samples used in this study.

**Supplemental Data Set 2.** Confirmation of wheat varieties in *Pst*-infected field samples.

**Supplemental Data Set 3.** Details of statistical analyses performed.

**Supplemental Data File 1.** Phylogenetic analysis of 96 *Pst*-infected wheat samples. Analysis was performed using 227,267,910 nucleotide sites and a maximum-likelihood model.

**Supplemental Data File 2.** Phylogenetic analysis of 96 *Pst*-infected wheat samples with bootstrap values. Analysis was performed using 227,267,910 nucleotide sites and a maximum-likelihood model.

## Supplementary Material

koab049_Supplementary_Data

## Notes

P.C-M. and D.G.O.S. wrote the manuscript; P.C-M. led and conducted the vast majority of experiments, analyzed the data and conducted all bioinformatic analysis; F.M. assisted with sample preparation and microscopy; P.E.D. with RNA extractions, E.W. with confocal microscopy, C.M.L. with infection assays, A.V.K. with cloning and plant growth, F.M., Y.M.L.M., and L.A.M-P. with RT-qPCR analysis; B.K., P.B., and L.H. performed metabolomic analysis; P.C-M. and D.G.O.S. conceived and designed experiments. All authors approved the final manuscript.

The author responsible for distribution of materials integral to the findings presented in this article in accordance with the policy described in the Instructions for Authors (https://academic.oup.com/plcell) is: Diane G.O. Saunders (Diane.Saunders@jic.ac.uk).
