# Supplementary material for: The branched-chain amino acid aminotransferase TaBCAT1 modulates amino acid metabolism and positively regulates wheat rust susceptibility
Source: Plant Cell. 2021 Feb 5;33(5):1728–47. doi: 10.1093/plcell/koab049 (PMC8254495; doi:10.1093/plcell/koab049)
Supplement: koab049_Supplementary_Data [file koab049_supplementary_data.zip › tpc.00530.2020-s06.pdf]

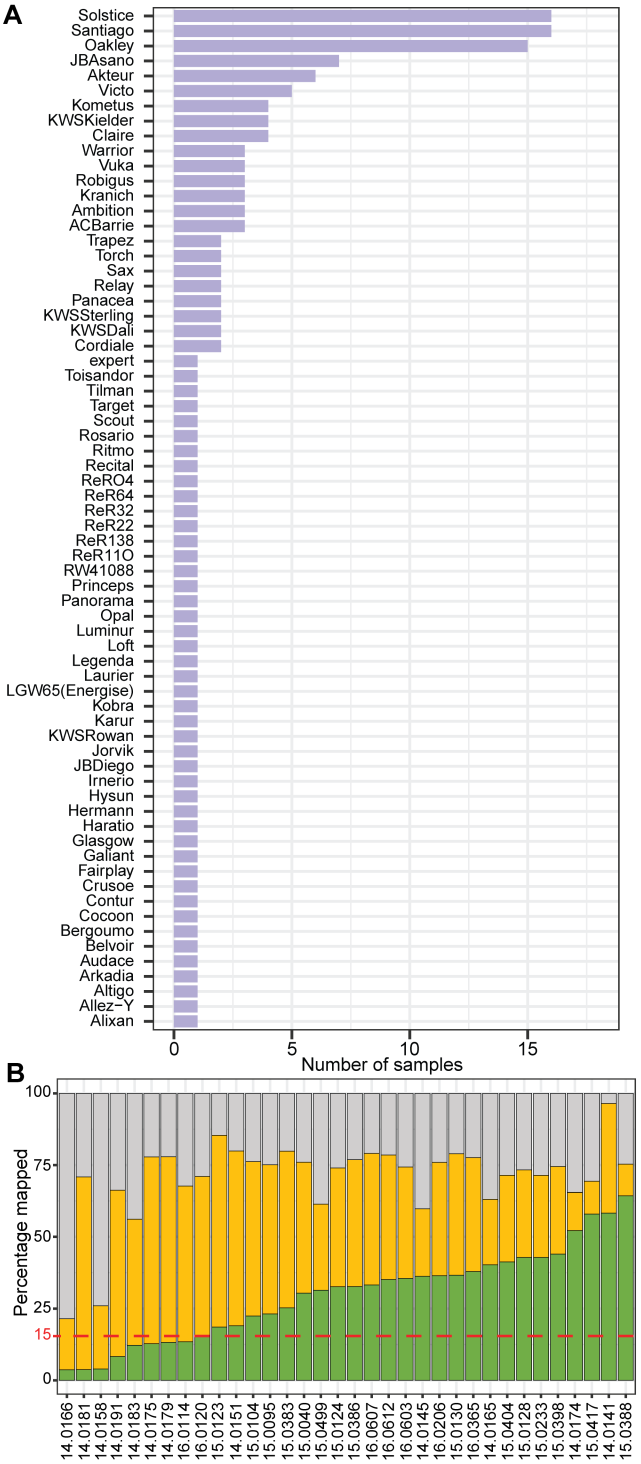

Supplemental Figure 1: *Pst*-infected wheat samples from the varieties Oakley, Solstice and Santiago selected for transcriptome analysis. (Supports Figure 1.) **A.** *Pst*-infected field samples were obtained from 68 known wheat varieties, with the largest number from the varieties Oakley, Solstice and Santiago. **B.** Following transcriptome sequencing (RNA-seq), a total of 21 *Pst*-infected samples from the varieties Oakley, Solstice and Santiago that met a minimum of 15% reads aligned to the wheat reference transcriptome and were confirmed as the recorded variety were selected for further analysis. Percentage of RNA-seq reads from *Pst*-infected wheat samples of the aforementioned varieties that mapped to the wheat reference transcriptome (IWGSC RefSeq v1.1; green), *Pst* reference (isolate PST-130; yellow) or were unmapped (grey).

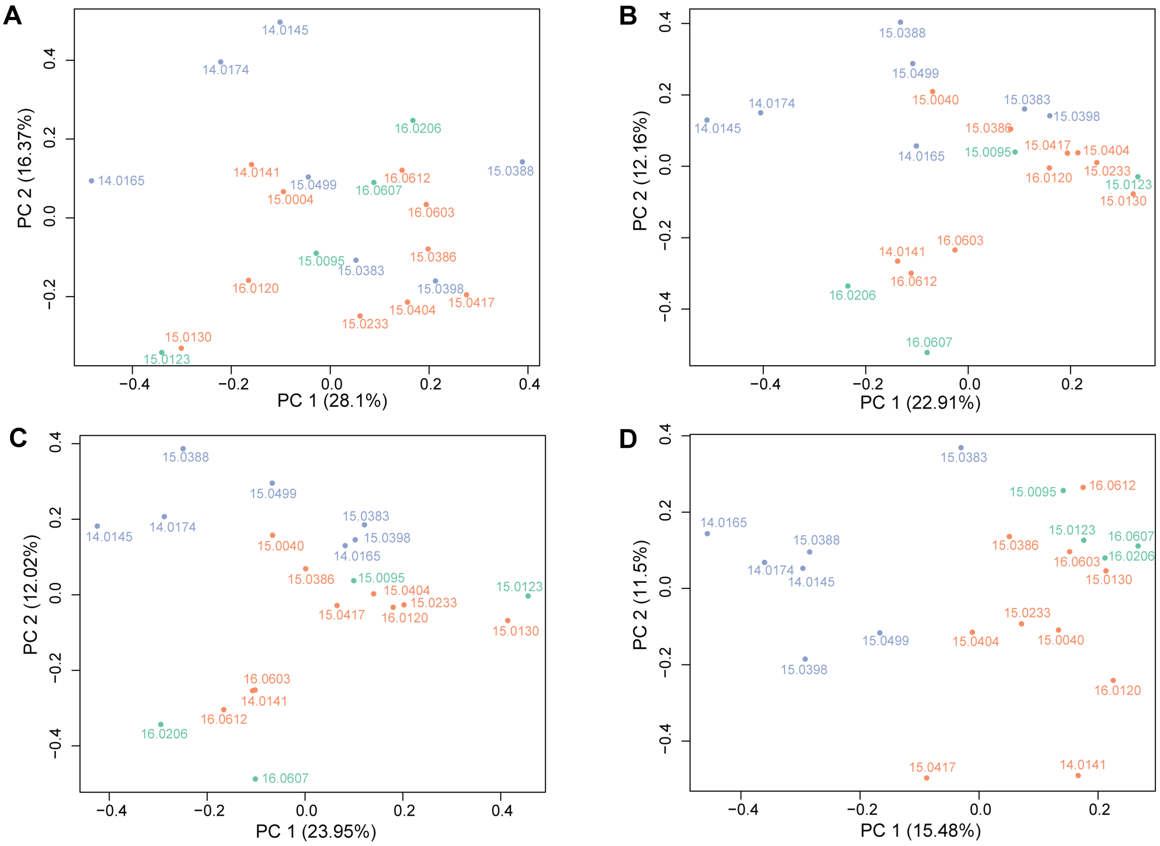

Supplemental Figure 2: Evaluation of normalization of transcript count data using principal component analysis from *Pst*-infected Oakley, Solstice and Santiago samples. (Supports Figure 1.) Between-sample normalization of read count data for 81,161 transcripts was undertaken to account for batch, library preparation and sequencing depth. Scatterplots of the first two principal components were generated for **A.** non-normalized counts, **B.** counts normalized by remove unwanted variation (RUV) using control genes, **C.** counts normalized by RUV using residuals and, **D.** counts normalized by RUV using replicate samples. Colors represent wheat variety (Solstice, Purple; Oakley, Green; Santiago, Orange).

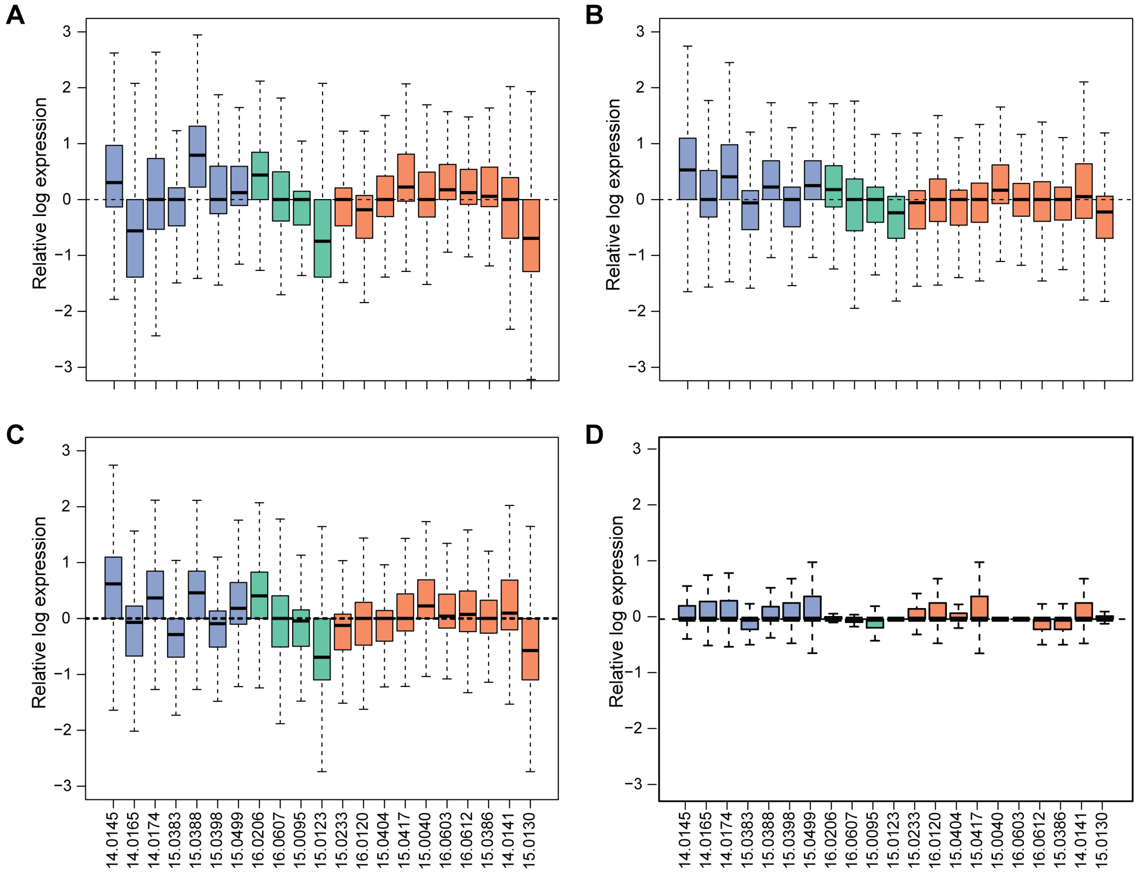

Supplemental Figure 3: Evaluation of normalization of transcript count data using relative log expression (RLE) values from *Pst*-infected Oakley, Solstice and Santiago samples. (Supports Figure 1.) Between-sample normalization of read count data for 81,161 transcripts was undertaken to account for batch, library preparation and sequencing depth. Boxplots of RLE values were generated for **A.** non-normalized counts, **B.** counts normalized by remove unwanted variation (RUV) using control genes, **C.** counts normalized by RUV using residuals and, **D.** counts normalized by RUV using replicate samples. Colors represent wheat variety (Solstice, Purple; Oakley, Green; Santiago, Orange); bars represent median values, boxes signify the upper (Q3) and lower (Q1) quartiles, and whiskers are located at 1.5 the inter-quartile range.

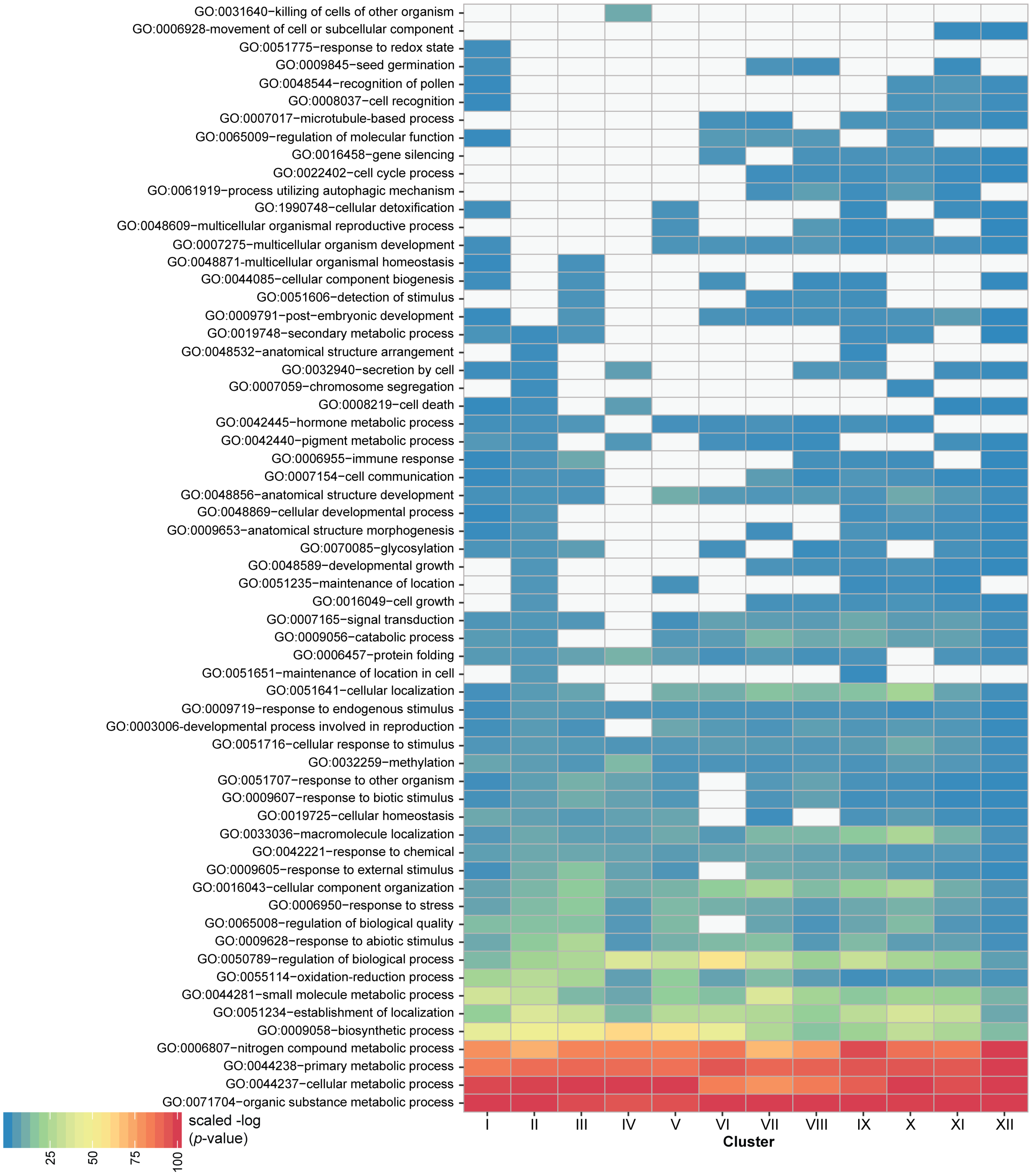

Supplemental Figure 4: Biological processes enriched in one or more of the 12 co-expression clusters. (Supports Figure 2.) A total of 61,700 differentially expressed transcripts were assigned to 12 co-expression clusters and gene ontology (GO) terms assigned to each gene where possible. Enrichment was determined based on Fisher's exact test and GO terms considered enriched in one cluster at *p*-values < 0.0001. Significant −log(*p*-values) are represented in a 0–100 scale (blue to red) and white fill represents terms not enriched in the given cluster.

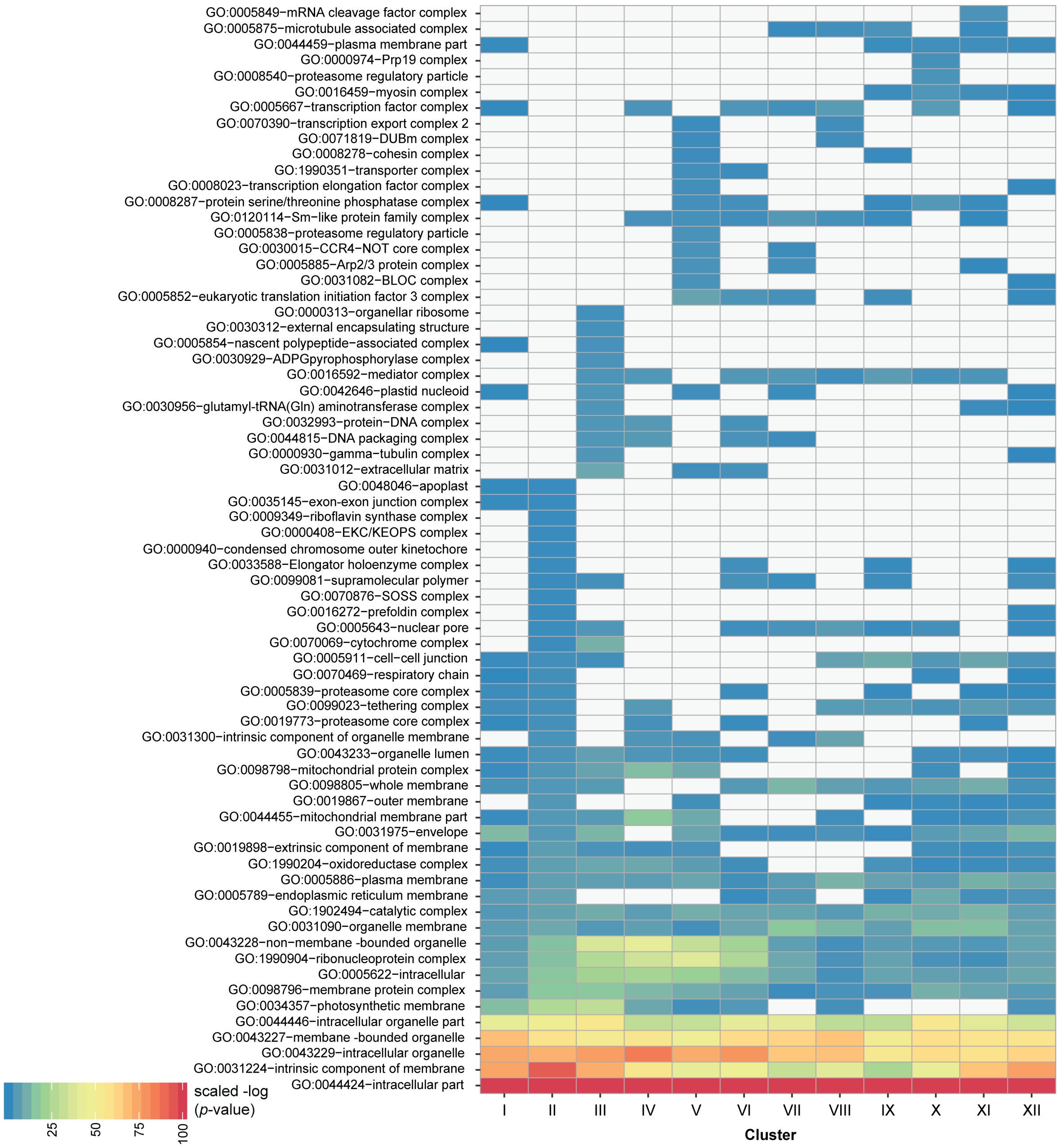

Supplemental Figure 5: Cellular components enriched in one or more of the 12 co-expression clusters. (Supports Figure 2.) A total of 61,700 differentially expressed transcripts were assigned to 12 co-expression clusters and gene ontology (GO) terms assigned to each gene where possible. Enrichment was determined based on Fisher's exact test and GO terms considered enriched in one cluster at *p*-values < 0.0001. Significant −log(*p*-values) are represented in a 0–100 scale (blue to red) and white fill represents terms not enriched in the given cluster.

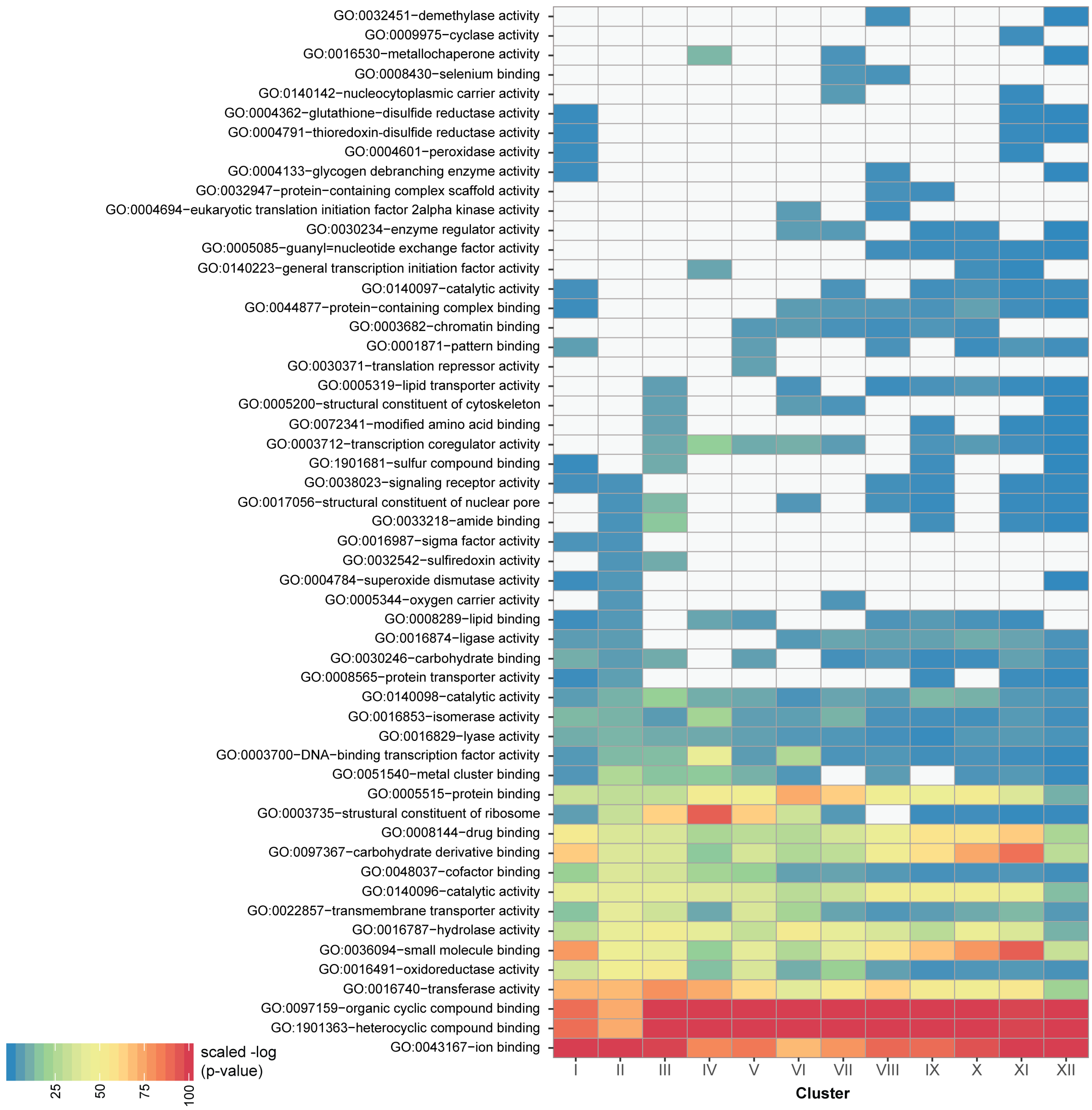

Supplemental Figure 6: Molecular functions enriched in one or more of the 12 co-expression clusters. (Supports Figure 2.) A total of 61,700 differentially expressed transcripts were assigned to 12 co-expression clusters and gene ontology (GO) terms assigned to each gene where possible. Enrichment was determined based on Fisher's exact test and GO terms considered enriched in one cluster at *p*-values < 0.0001. Significant −log(*p*-values) are represented in a 0–100 scale (blue to red) and white fill represents terms not enriched in the given cluster.

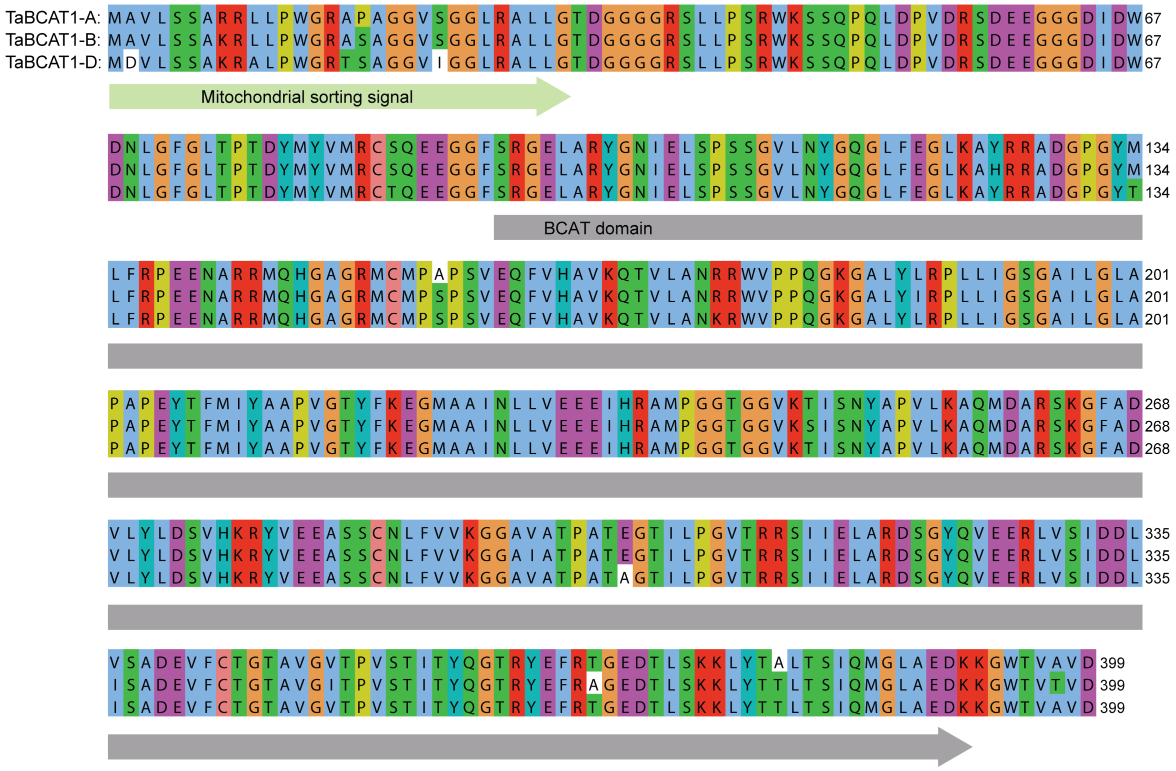

Supplemental Figure 7: Multiple sequence alignment of the protein sequences of the three *Triticum aestivum* homeologs of TaBCAT1. (Supports Figure 3.) An N-terminal mitochondrial sorting signal was identified (amino acids 1–30; green arrow) and BCAT domain (amino acids 93–391; grey arrow). Amino acids are coded in Clustalx color format.

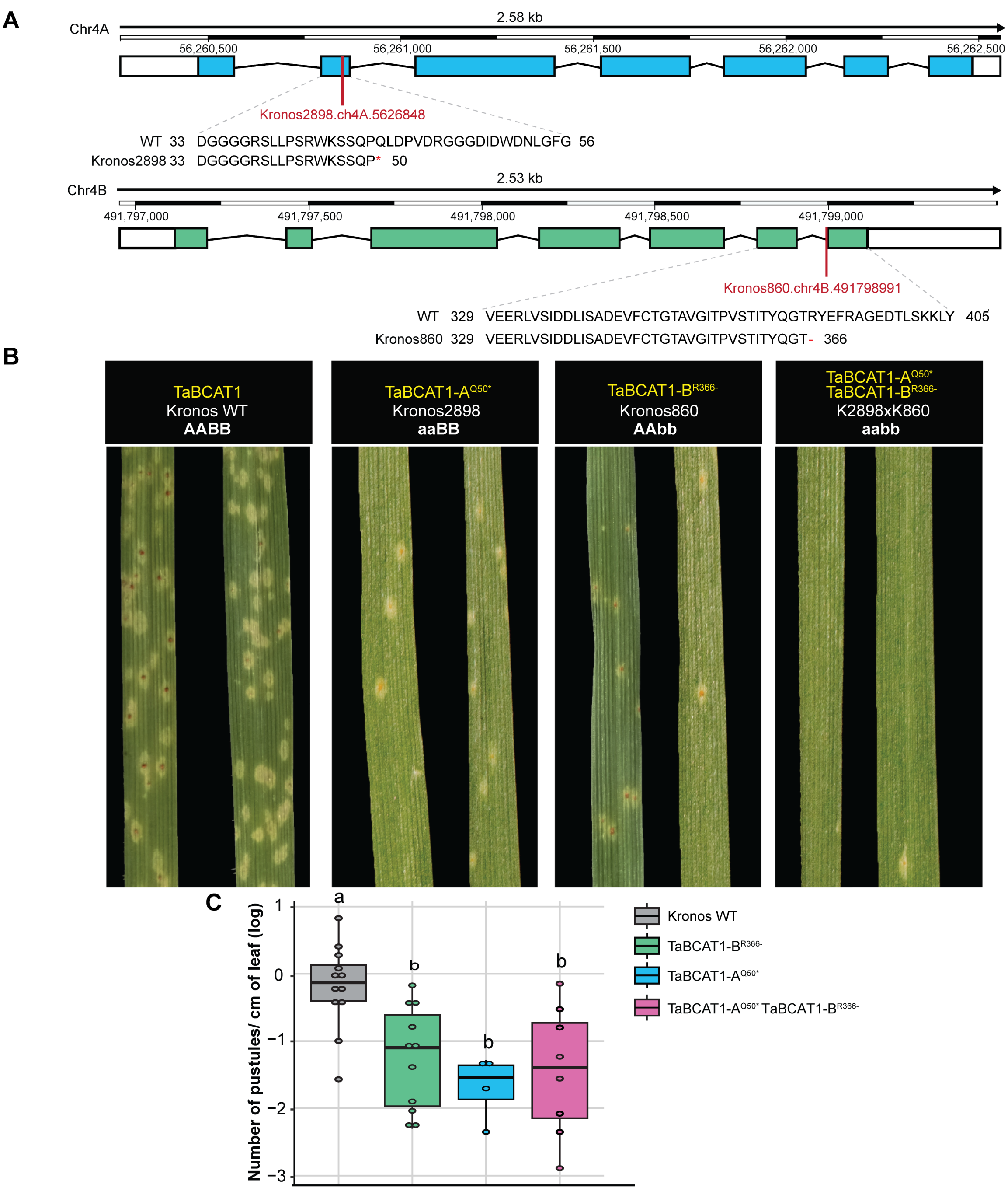

Supplemental Figure 8: *TaBCAT1* disruption mutants display a significant reduction in susceptibility to *Pgt*. (Supports Figure 4.) **A.** *TaBCAT1* is located on chromosome 4 and loss-of-function tetraploid Kronos targeting local lesions in genomes (TILLING) mutants were identified. For the A genome, a mutant that encoded an early stop codon mutation at amino acid 50 (line Kronos2898) was identified and for the B genome, a mutant with a splice acceptor variant in the last intron, predicted to result in a protein truncation at amino acid 366 (line Kronos860). Chr, chromosome; kb, kilobase; WT, wild type. **B.** A reduction in the total number of lesions in both single mutants (TaBCAT1-A^{Q50*} and TaBCAT1-B^{R366-}) and the double mutant (TaBCAT1-A^{Q50*} TaBCAT1-B^{R366-}) was observed when lines were infected with *Pgt* isolate UK-01. Negative control, Kronos wild type (WT). Images were captured 16 days post-inoculation (dpi). **C.** The number of *Pgt* pustules observed per leaf cm was significantly reduced in *TaBCAT1* disruption mutants at 16 dpi. Letters indicate significant differences determined using Duncan's multi range test ($p < 0.05$). WT plants $n = 12$, TaBCAT1-B^{R366-} $n = 11$, TaBCAT1-A^{Q50*} $n = 4$ and TaBCAT1-A^{Q50*} TaBCAT1-B^{R366-} $n = 8$. Bars represent median values, boxes signify the upper (Q3) and lower (Q1) quartiles, and whiskers are located at 1.5 the inter-quartile range.

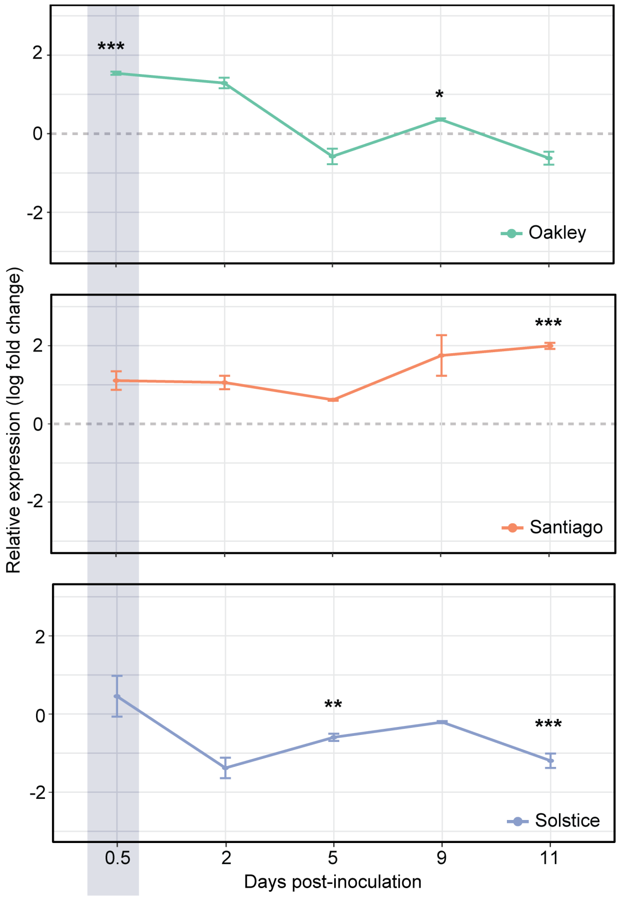

Supplemental Figure 9: *TaBCAT1* expression early during *Pst* infection is linked to wheat varietal susceptibility. (Supports Figure 5.) A controlled time-course of infection was carried out with *Pst* isolate 13/14 and wheat varieties Oakley, Solstice and Santiago. During *Pst* infection, *TaBCAT1* expression was significantly higher in the most susceptible variety Oakley. The initial higher level of *TaBCAT1* expression then decreased substantially by 5 dpi. Two independent leaves from the same plant were pooled and 3 independent plants analyzed for *TaBCAT1* expression at each time point between *Pst*-infected and mock-inoculated plants. Asterisks denote statistically significant differences (***: $p < 0.005$, **: $p < 0.01$, *: $p < 0.05$; 2-tailed *t*-test). Error bars represent standard deviation.

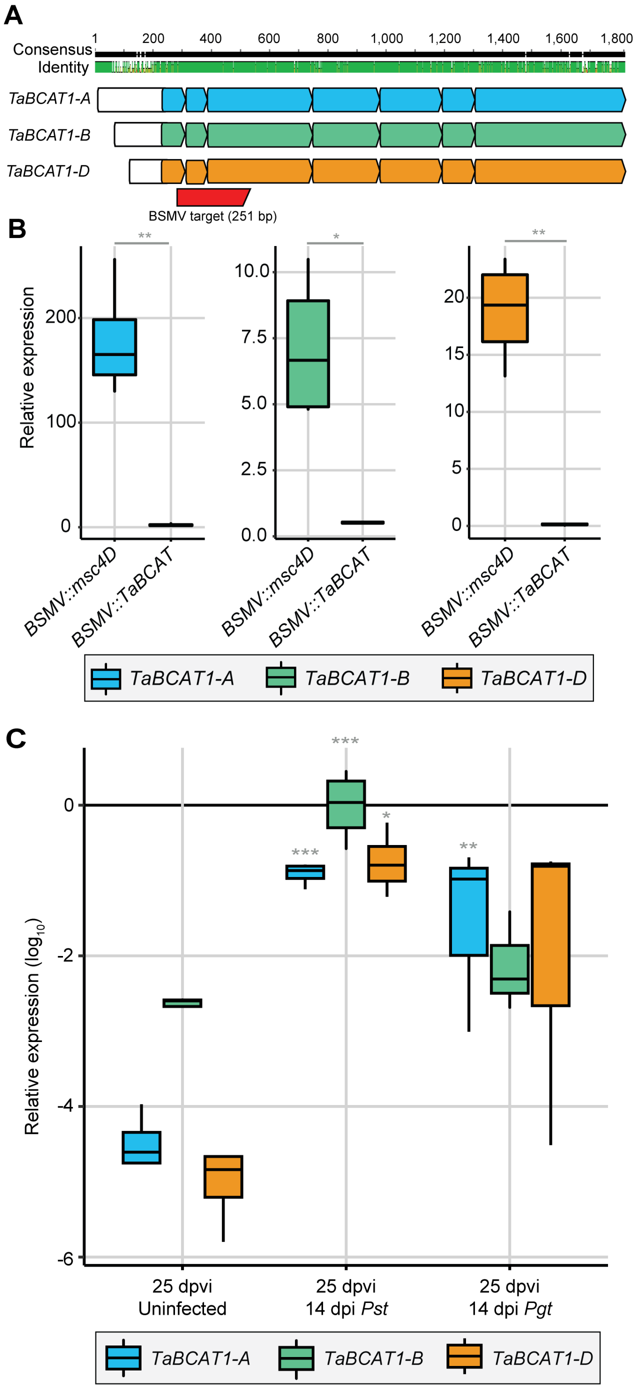

Supplemental Figure 10: Virus-induced gene silencing of *TaBCAT1*. (Supports Figure 6.) **A.** A 251-bp fragment of the *TaBCAT1* gene was designed to target the three homoeologous copies of the gene simultaneously for silencing. **B.** Expression of the three *TaBCAT1* homoeologs (*TaBCAT1-A*, *TaBCAT1-B* and *TaBCAT1-D*) was significantly reduced 25 days post-viral inoculation. Levels of *TaBCAT1* expression were measured by RT-qPCR in plants silenced with *BSMV::TaBCAT1* and compared to the negative control where BSMV*::msc4D* was utilized as a viral infection control. A total of 4 samples each were evaluated for *BSMV::msc4D* and *BSMV::TaBCAT1*. **C.** Silencing of *TaBCAT1* was alleviated after *Pst* or *Pgt* inoculation and *TaBCAT1* expression returned to WT levels. Plants were infected with *Pst* isolate 13/14 or *Pgt* isolate UK-01 and expression levels assessed 25 dpvi using RT-qPCR. Asterisks denote statistically significant differences (***: $p < 0.001$, **: $p < 0.01$, *: $p < 0.05$; 2-tailed *t*-test). Bars represent median values, boxes signify the upper (Q3) and lower (Q1) quartiles, and whiskers are located at 1.5 the inter-quartile range.

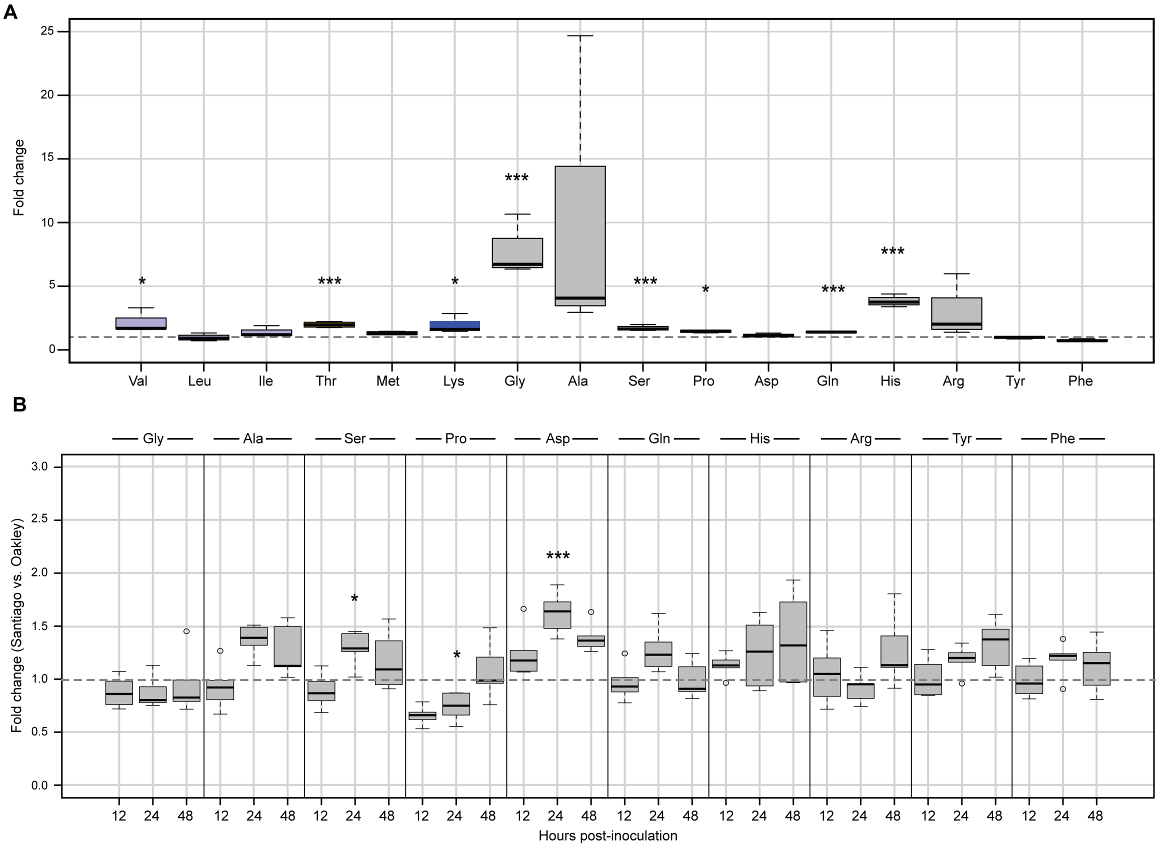

Supplemental Figure 11: Levels of the BCAAs Val and Ile are enhanced in the *TaBCAT1* disruption mutant and of the remaining amino acids, Ser and Asp levels were modulated dependent on the level of *Pst* susceptibility during infection. (Supports Figure 7.) **A.** Levels of free amino acids were generally moderately higher in the *TaBCAT1* disruption mutant line (n = 4) when compared to the Kronos wild type (n = 5). This included the BCAAs Val and Ile and Asp-derived amino acids Thr, Met and Lys. **B.** Excluding the BCAAs and Asp-derived amino acids, Ser and Asp were significantly increased at 24 hours post-inoculation (hpi) in the resistant variety Santiago (n = 5) during *Pst* infection (isolate F22) when compared to the susceptible variety Oakley (n = 5). Amino acid levels were assessed at 12, 24 and 48 hpi. Asterisks denote statistically significant differences (***: $p < 0.001$, *: $p < 0.05$; 2-tailed t-test). Bars represent median values, boxes signify the upper (Q3) and lower (Q1) quartiles, and whiskers are located at 1.5 the inter-quartile range.

Supplemental Table 1: Seedling infection assays illustrate that the three wheat varieties Oakley, Solstice and Santiago have different levels of susceptibility to the *Pst* isolates F22 and 13/14. Each wheat variety was subjected to *Pst* infection with two isolates (F22 and 13/14) and infection types (IT) were recorded 12 days post-inoculation following the 0–4 scale (McIntosh et al., 1995). A total of 5 independent plants considered as biological replicates (rep.) were analyzed.

Wheat variety	*Pst* isolate	Rep. 1	Rep. 2	Rep. 3	Rep. 4	Rep. 5	Average	Host response
Oakley	F22	4	3	4	4	3	3.6	Susceptible
Santiago	F22	0	0	1	0	1	0.4	Resistant
Solstice	F22	2	3	3	2	2	2.4	Moderately resistant
Oakley	13/14	4	3	4	4	3	3.6	Susceptible
Santiago	13/14	3	3	2	3	3	2.8	Moderately susceptible
Solstice	13/14	4	3	4	4	4	3.8	Susceptible

Supplemental Table 2: RT-qPCR primer sequences and efficiencies.

Gene name	Gene ID Refseq v1.1	Forward primer	Reverse primer	Efficiency	Reference
***TaBCAT1* (common)**	TraesCS4A02G059800, TraesCS4B02G235400, TraesCS4D02G236800	GGTGTCGACCATCACCTACC	CTTCTTCGACAGCGTGTCCT	100.60%	This study
TaBCAT1-A	TraesCS4A02G059800	GGTGTCGACCATCACCTACC	CTTCTTCGACAGCGTGTCCT	105.19%	This study
TaBCAT1-B	TraesCS4B02G235400	GGGACAAGGTACGAGTTCAGG	CCGTCCATCCCTTCTTGTCC	100.54%	This study
TaBCAT1-D	TraesCS4D02G236800	ACGAGTTCAGGACCGGAGAA	AACCGTCCATCCTTTCTTGTCC	104.36%	This study
TaPR1	TraesCS5A02G183300	CTGGAGCACGAAGCTGCAG	CGAGTGCTGGAGCTTGCAGT	102.21%	(Molina et al 1999)
TaPR2	TraesCS5A02G017900	CTCGACATCGGTAACGACCAG	GCGGCGATGTACTTGATGTTC	110.68%	(Ray et al 2003)
TaPR3	TraesCS2B02G125200	AGAGATAAGCAAGGCCACGTC	GGTTGCTCACCAGGTCCTTC	98.05%	(Desmond et al 2006)
TaPR4	TraesCS3B02G529700	CGAGGATCGTGGACCAGTG	GTCGACGAACTGGTAGTTGACG	98.58%	(Bertini et al 2003)
TaPR5	TraesCS3A02G517100	ACAGCTACGCCAAGGACGAC	CGCGTCCTAATCTAAGGGCAG	94.41%	(Kuwabara et al 2002)
TaPR9	TraesCS1A02G203700	GAGATTCCACAGATGCAAACGAG	GGAGGCCCTTGTTTCTGAATG	90.85%	(Pritsch et al 2000)
UBC4 (Reference gene)	TraesCS4A02G414200 TraesCS4B02G314900 TraesCS4D02G312000	ACAAGGTCGAGACGGTGAAC	GTAAGGATACGCATCGGGCA	104.31%	(Borrill et al. 2016)

Supplemental Table 3: Probability of mitochondrial localization for TaBCAT1 homoeologous proteins. The programs MitoProt and TargetP were used to search the N-terminus for signatures typical of a mitochondrial targeting and cleavage sequence. Values represent percentage probability of containing an N-terminal mitochondrial targeting sequence.

Protein copy	Mitoprot	TargetP
**TaBCAT1-A**	0.9279	0.8539
**TaBCAT1-B**	0.9579	0.7847
**TaBCAT1-D**	0.8292	0.1660

Supplemental Table 4: KASP primers to genotype TILLING lines selected. Primers were designed with additional standard FAM or HEX compatible tails. WT primers (A) were designed with FAM tails (5′ GAAGGTGACCAAGTTCATGCT 3′) and MUT primers (B) with HEX tails (5′ GAAGGTCGGAGTCAACGGATT 3′).

Refseq v1.1 ID	Line	WT primer (A)	MUT primer (B)	Common primer
TraesCS4A02G059800	Kronos2898	aagtcgtcgcagccgC	aagtcgtcgcagccgT	agccattggcgatcgAtcAaA
TraesCS4B02G235400	Kronos860	gtgctgagatatgtgcgttcaG	gtgctgagatatgtgcgttcaA	ttcttcgacaGcgtgtcctc
